# Variation in seed longevity among diverse Indica rice varieties

**DOI:** 10.1093/aob/mcz093

**Published:** 2019-06-10

**Authors:** Jae-Sung Lee, Marlina Velasco-Punzalan, Myrish Pacleb, Rocel Valdez, Tobias Kretzschmar, Kenneth L McNally, Abdel M Ismail, Pompe C Sta Cruz, N Ruaraidh Sackville Hamilton, Fiona R Hay

**Affiliations:** 1 International Rice Research Institute, Los Baños, College, Laguna, Philippines; 2 Institute of Crop Science, University of the Philippines Los Baños, College, Laguna, Philippines; 3 Southern Cross Plant Science, Southern Cross University, Lismore, NSW, Australia; 4 Department of Agroecology, Aarhus University, Forsøgsvej, Slagelse, Denmark

**Keywords:** Genebank, GWAS, Indica variety group, *Oryza sativa* L, rice, seed longevity, seed storage, viability monitoring

## Abstract

**Background and Aims:**

Understanding variation in seed longevity, especially within closely related germplasm, will lead to better understanding of the molecular basis of this trait, which is particularly important for seed genebanks, but is also relevant to anyone handling seeds. We therefore set out to determine the relative seed longevity of diverse Indica rice accessions through storage experiments. Since antioxidants are purported to play a role in seed storability, the antioxidant activity and phenolic content of caryopses were determined.

**Methods:**

Seeds of 299 Indica rice accessions harvested at 31, 38 and 45 d after heading (DAH) between March and May 2015 and differing in harvest moisture content (MC) were subsequently stored at 10.9 % MC and 45 °C. Samples were taken at regular intervals and sown for germination. Germination data were subjected to probit analysis and the resulting parameters that describe the loss of viability during storage were used for genome-wide association (GWA) analysis.

**Key Results:**

The seed longevity parameters, *K*_i_ [initial viability in normal equivalent deviates (NED)], −*σ*^−1^ (*σ* is the time for viability to fall by 1 NED in experimental storage) and *p*_50_ [time for viability to fall to 50 % (0 NED)], varied considerably across the 299 Indica accessions. Seed longevity tended to increase as harvest MC decreased and to decrease as harvest MC increased. Eight major loci associated with seed longevity parameters were identified through GWA analysis. The favourable haplotypes on chromosomes 1, 3, 4, 9 and 11 enhanced *p*_50_ by ratios of 0.22–1.86.

**Conclusions:**

This is the first study to describe the extent of variation in σ within a species’ variety group. *A priori* candidate genes selected based on rice genome annotation and gene network ontology databases suggested that the mechanisms conferring high seed longevity might be related to DNA repair and transcription, sugar metabolism, reactive oxygen species scavenging and embryonic/root development.

## INTRODUCTION

Orthodox seeds can remain viable for many years if stored at low moisture content (MC) and low temperature (Bewley and Black, 1994). This ‘storability’ or ‘longevity’ is exploited as a means of *ex situ* conservation of biodiversity in seed-/genebanks. However, the length of time that seeds can survive varies among species (Justice and Bass, 1978; Walters *et al.*, 2005; Probert *et al.*, 2009; Merritt *et al.*, 2014). It also varies among seed lots within a species and among the individual seeds within a seed lot. The variation in seed longevity within a seed lot is usually described by fitting a normal distribution of seed deaths over time, the basis of the seed viability equations (Ellis and Roberts, 1980). Collecting fully matured seeds is critical to ensuring maximum longevity during storage (Hay and Smith, 2003), but how the seeds are dried, processed and held before storage and the length of time that these steps take also affect subsequent longevity (Probert *et al.*, 2007; Whitehouse *et al.*, 2015). Understanding why some seeds remain alive for a long time and others, under the same conditions, for a short time, has been a fundamental question in seed biology. As well as being important for predicting viability during storage, it is important in terms of managing seed vigour. Seed lots with high viability are likely to have high vigour; reduced vigour is an early symptom of seed ageing.

A number of seed constituents have been purported to play a role in conferring stability in storage, including sugars, proteins and antioxidants. High levels of raffinose family oligosaccharides, or high levels relative to the amount of sucrose, have been correlated with seed longevity (Bailly *et al.*, 2001; ElSayed *et al.*, 2014) and De Souza Vidigal *et al.* (2016) concluded that galactinol, an intermediate metabolite in the biosynthesis of raffinose oligosaccharides, can be used as a marker of longevity among different varieties of Brassicaceae or tomato. However, some authors concluded that proteins must contribute to the stabilizing effect of sugars, in particular proteins that are expressed late in seed development (Chatelain *et al.*, 2012). Repair processes that are initiated during the early stages of seed germination and which overcome the damage to macromolecules incurred during ageing have also been identified as key to ‘longevity’ (Waterworth *et al*., 2010, 2015). Much of the damage that occurs during seed ageing is attributed to the generation and action of reactive oxygen species; hence, the ability to neutralize these reactive oxygen species through an effective antioxidant system is also important (Noctor and Foyer, 1998; Bailly, 2004; Sattler *et al.*, 2004; Nguyen *et al.*, 2015). Given these different hypotheses relating to seed longevity, and the plasticity of the trait, it is not surprising that a complex network of putative longevity-related genes has been reported (Righetti *et al.*, 2015).

Other work relating genes and seed longevity has taken a quantitative genetics approach. Through genetic linkage mapping using inbred line or recombinant inbred line maize populations, a number of quantitative trait loci (QTLs) and candidate genes associated with seed longevity under natural (low MC and low temperature) and artificial ageing (high MC and high temperature) conditions have been identified (Revilla *et al.*, 2009; Han *et al.*, 2014). A genome-wide association (GWA) study for a diverse barley panel discovered over 100 genetic markers correlated with seed longevity (Nagel *et al.*, 2015). Sano *et al.* (2017) conducted QTL analysis and RNA sequencing for *Arabidopsis* recombinant inbred lines and identified a negative correlation between brassinosteroids and seed longevity. Relatively few studies have reported candidate genes involved in rice (*Oryza sativa*) seed longevity. Most have been QTL studies using biparental mapping populations, leading to the identification of large chromosome regions rather than candidate genes. The marker interval of the most well-defined QTL spanned a region of 0–72 cM on chromosome 9 (Miura *et al.*, 2002; Sasaki *et al.*, 2005; Xue *et al.*, 2008; Li *et al.*, 2012). Sasaki *et al.* (2015) fine-mapped this region using near-isogenic lines and identified the candidate gene, *TPP7*, a trehalose-6-phosphate phosphatase known to be involved in anaerobic germination tolerance (Kretzschmar *et al.*, 2015). However, these studies on rice seed longevity were limited to genes where the two parents (usually Japonica × Indica) differ. Different studies also used different ageing/storage conditions and measures of ‘longevity’ (Hay *et al.*, 2019). Furthermore, they did not take into account variation in the timing of harvest maturity, which is known to have an effect on subsequent seed longevity (Hay *et al.*, 2015). Compared with conventional biparental QTL mapping, GWA has several advantages: (1) it can identify a range of genes with allelic variation among a large number of diverse accessions; and (2) there is typically higher resolution of QTLs, allowing the direct identification of candidate genes without need for further fine mapping (Collard *et al.*, 2008). Although a GWA panel could span different variety groups, haplotypes are often subpopulation-specific (Famoso *et al.*, 2011). Consequently, restricting a panel to a single variety group increases the power of QTL detection for genes that are polymorphic only within that variety group (Al-Tamimi *et al.*, 2016; McCouch *et al.*, 2016).

The International Rice Genebank at the International Rice Research Institute conserves over 127 000 accessions of *Oryza sativa*, *O. glaberrima* and their wild relatives. With such a large number of accessions, routine viability monitoring involves thousands of germination tests each year, requiring the destructive use of seeds, and considerable time and money. Appropriate adjustment of monitoring intervals based on known variation in seed longevity across genotypes is one way in which genebank management can be improved. The aims of this study were to: (1) characterize the survival curves of seeds of diverse Indica rice accessions; and (2) identify loci/candidate genes associated with seed longevity in Indica rice through GWA analysis, taking into account the effects of seed maturity and harvest moisture content.

## MATERIALS AND METHODS

### Plant materials

A diverse panel of 299 Indica rice accessions from the 3,000 Rice Genomes Project (2014) were used (Supplementary Data Tables S1 and S2; Supplementary Data File S1). Seed samples were taken out of genebank storage in December 2014. After breaking dormancy (50 °C for 7 d), seeds were germinated in seedling plots and 20-d-old seedlings were transplanted to the Zeigler Experiment Station, with 200 mm spacing of plants within and between rows; 28 kg ha^−1^ NPK (14-14-14) was applied during field preparation. Nitrogen (20 kg ha^−1^) was applied 30 and 50 d after transplanting. Insect, disease and weed control was consistent with standard International Rice Research Institute procedures. A total of 12 plants per sample (seed lot) were planted and harvested as bulk. The day when 80 % of plants had completed heading was considered the heading date. Seeds were harvested at 31, 38 and 45 d after heading (DAH) and threshed. Harvest MC was determined by weighing three replicate samples of ground seeds before and after 2 h of oven-drying at 130 °C followed by 1 h at room temperature (ISTA, 2018). Blown and sorted seed lots were dried at 15 °C, 15 % RH. For each seed lot, three samples of 100 dried seeds were weighed to determine 100-seed weight. The bulk of each seed lot was then sealed inside aluminium foil bags and stored at −20 °C.

### Seed longevity phenotyping

A standard protocol was used for the seed storage experiments (Hay *et al*., 2015, 2019; Whitehouse *et al.*, 2015), done in batches of ten accessions. Samples of 60 seeds for each accession × harvest maturity were equilibrated at 60 % RH, 25 °C in a climate test chamber (Model VC3 0034-M; Vötschtechnik, Germany) and then transferred to laminated aluminium foil bags, which were immediately heat-sealed and placed at 45 °C. Samples were taken at 7-d intervals from 0 to 63 d and tested for ability to germinate. For each sample, 30 seeds were placed on two layers of Whatman No. 1 filter paper with 7 mL of distilled water in each of two 90-mm-diameter Petri dishes. Seeds were germinated at 30 °C with 12 h light per day and scored daily up to 21 d after sowing (seeds sampled at 0 d storage) or at 5, 9, 14 and 21 d after sowing (all other samples). Two or three sets of three samples each were also used for monitoring MC before, during and/or at the end of storage.

Germination data for each seed lot were analysed by probit analysis in GenStat v. 18 (VSN International, Hemel Hempstead, UK), thereby fitting the Ellis and Roberts (1980) viability equation:

$$\begin{array}{l} v=K_{i}-{}^{\;p}╱{}_{\sigma }\; \end{array}$$ (1)

where *v* is the viability in normal equivalent deviates (NED) after *p* days storage, *K*_i_ is the initial viability (NED) and *σ* is the time it takes for viability to fall by 1 NED. Thus, −*σ*^−1^ is the slope of the transformed survival curve. The time for viability to fall to 50 %, *p*_50_, was also estimated. For seed lots for which germination initially increased or was maintained at ~100 %, only the data covering the period when viability was declining were included in the analysis.

### Determination of antioxidant and phenol content

Antioxidants in the caryopses of the seed lot with greatest longevity (*p*_50_) as determined from the seed storage experiment were screened following Shimada *et al.* (1992) and Dewanto *et al.* (2002). Ground and then freeze-dried caryopses (0.3 g) were extracted with 6 mL of 80 % MeOH at 40 °C for 3 h. To quantify free radical scavenging activity, the extract was reacted with 1,1-diphenyl-2-picrylhydrazyl (DPPH; Sigma Co. Singapore) at 25 °C for 30 min in darkness, then scavenging activity was determined by maximum absorption at 518 nm using a DU 800 UV/Vis spectrophotometer (Beckman Coulter, Germany). The other sample extract was mixed with 50 % Folin-Ciocalteu’s phenol reagent, 2 N (Sigma Aldrich, USA) plus 2 % Na_2_CO_3_ solution at 25 °C for 30 min. Total phenolic content was determined by maximum absorption at 725 nm.

### Association analysis

Descriptive statistics of individual traits and correlations between seed longevity and related traits were analysed using STAR 2.0.1 (International Rice Research Institute, Philippines). Genome-wide association analysis was conducted in TASSEL 5.2.7 (Bradbury *et al.*, 2007) using the 18 million single-nucleotide polymorphism (SNP) base set of the 3,000 Rice Genomes Project (Wang *et al.*, 2018). After filtering SNPs for 20 % missing data and minor allele frequencies (<5 %), 988 k SNPs were retained for association analysis. A mixed linear model (MLM) with kinship matrix and general linear model (GLM) with principal components analysis (PC_5_) were applied. Based on quantile–quantile plots for the GLM, there was considerable overestimation (false positives), which may mislead the candidate gene selection. When PC_5_ was included in the GLM, the estimation of quantile–quantile plots was improved. Plot-peaks above the threshold (*P* < 9.99E−06) where the –log_10_*P* values were not linear in the quantile–quantile plot and detected in major loci of both MLM and GLM with PC_5_ were considered to be associated with the traits.

## RESULTS

### Variation in heading date, seed harvest moisture content and 100-seed weight

**Fig. 1.**
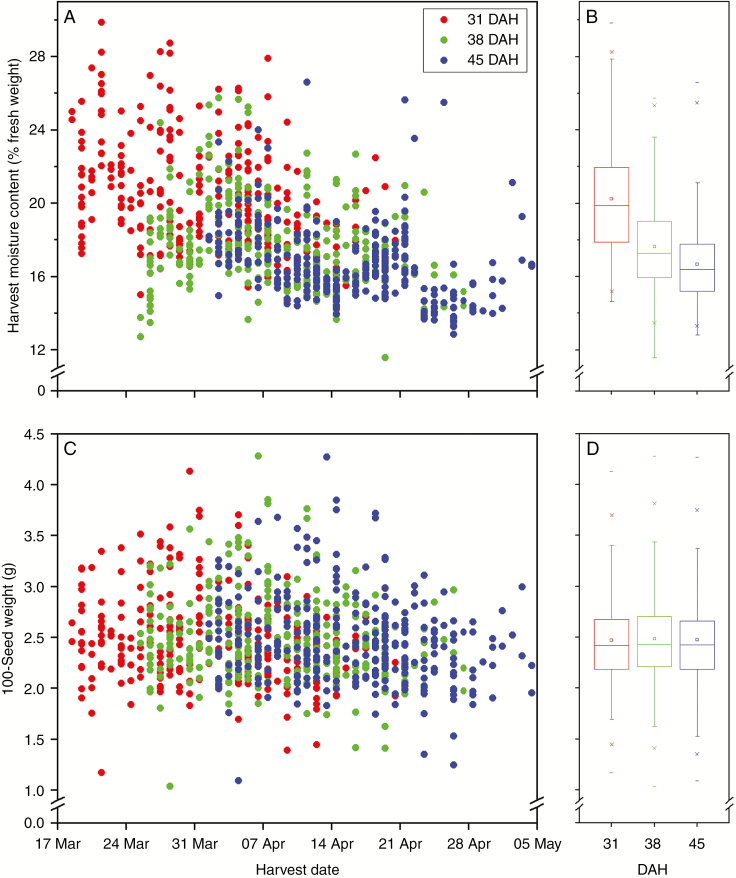
Changes in seed harvest moisture content (MC) and 100-seed weight over the harvesting period for 299 Indica rice accessions. Seeds were harvested at 31, 38 and 45 days after heading (DAH). Panels (B) and (D) are box plots of the data shown in (A) and (C), to summarize the changes in harvest MC and 100-seed weight for the different harvests. In (B) and (D) the square symbol is the mean, the box spans the 25th and 75th percentiles, the whiskers represent the 5th and 95th percentiles, the crosses the 1st and 99th percentiles, and the dashes the minimum and maximum values, respectively.

Length of time from sowing to 80 % heading ranged between 67 and 100 d, with a mean of 82 d. Due to this variation in heading date, there was also variation in the dates of harvest (Fig. 1). For seeds harvested at 31 DAH, the first accessions were harvested on 19 March and the last accessions were harvested on 21 April. The last harvests of 45 DAH seeds were on 5 May. Seed harvest MC (% fresh weight) across all seed lots ranged between 11.57 and 25.74 %, but showed a decreasing trend with harvest date and maturity (Fig. 1A, B). Across accessions, the mean harvest MC of 31 and 38 DAH seeds was significantly greater than that of 38 and 45 DAH seeds, respectively (paired *t*-test, *P* < 0.001). The 100-seed weight ranged between 1.04 and 4.28 g (Fig. 1C, D; Table 1). Across accessions, the 100-seed weight of seeds harvested at 38 DAH (2.487 g) was significantly higher than that of seeds harvested at 31 DAH (2.470 g; *P* = 0.03) or 45 DAH (2.475 g; *P* = 0.016).

**Table 1.** Traits measured for seeds of 299 Indica rice accessions

Trait	Range	Mean (s.d.)	Coefficient of variation
100-seed weight (g) (seeds dried at 15 % RH, 15 °C)	1.04 to 4.28	2.49 (0.42)	16.80
*K*_i(38DAH)_ (NED)	0.12 to 5.84	2.70 (1.04)	38.59
−*σ*_(38DAH)_^−1^ (d^−1^)	−0.50 to −0.03	−0.16 (0.08)	−47.67
*p*_50(38DAH)_ (d)	1.03 to 59.12	19.91 (10.04)	50.44
*K*_i(max)_ (NED)	0.53 to 6.29	3.32 (1.08)	32.44
−*σ*_(max)_^−1^ (d^−1^)	−0.50 to −0.03	−0.16 (0.08)	−48.38
*p*_50(max)_ (d)	5.41 to 59.12	24.24 (10.75)	44.32
Antioxidant activity (%) in caryopses^1^	30.30 to 91.50	68.82 (10.48)	15.21
Phenolic content (μg g^–1^) of caryopses^1^	18.6 to 3268	599.0 (470.8)	78.6

The seed longevity parameters *K*_i_, −*σ*^–1^ and *p*_50_ are shown for seeds harvested at 38 days after heading (DAH) and for the seed lot with maximum (max) *p*_50_.

^1^Results only available for 240 accessions.

### Seed longevity

The mean MC during seed storage (across all seed lots and sampling times) was 11.13 %; the range across batches was between 10.77 and 11.66 %. The seeds did not consistently lose or gain moisture during the seed storage experiment, or differ in MC within an accession depending on maturity (data not shown). The plots of ability to germinate following different periods of storage were typically sigmoid (Supplementary Data Fig. S1). When fitting eqn (1) to the seed storage data, it was possible to constrain the fitted survival curves for the different seed maturities to a common slope (−*σ*^−1^) without a significant increase in residual deviance (approximate *F*-test, *P >* 0.05) for 95 accessions; for the other accessions, parameters could not be constrained. For most accessions, there was an increase in *K*_i_ and the gradient of the survival curve decreased (i.e. *σ* increased) between 31 and 38 DAH; hence, *p*_50_ increased. For 69 accessions, 38 DAH was the optimum harvest time for maximum *p*_50_, but for 227 seed lots seed longevity (*p*_50_) was greatest for seeds harvested at 45 DAH.

**Fig. 2.**
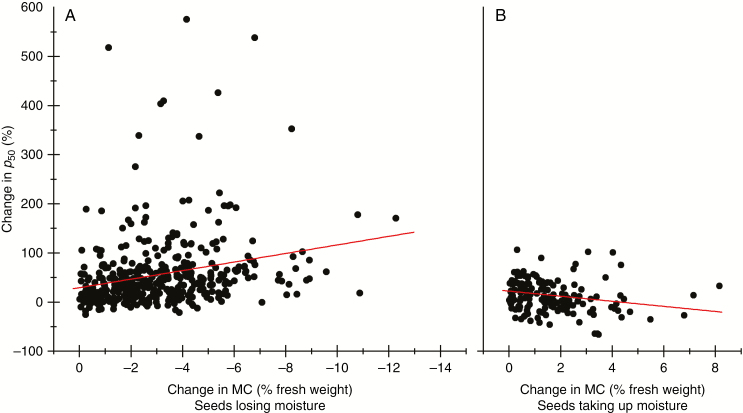
Relationships between the change in longevity (time for viability to fall to 50 % during experimental storage, *p*_50_) and change in harvest MC for Indica rice seeds that either (A) lost moisture between sequential harvests [i.e. between 31 and 38 days after heading (DAH) or between 38 and 45 DAH] or (B) gained moisture. The change in *p*_50_ was calculated as a proportion of the *p*_50_ of the first harvest (i.e. 31 or 38 DAH, respectively). The red lines show the results of linear regression analysis. Seeds were stored at 45 °C and 10.77–11.66 % moisture content.

For seeds that were losing moisture between sequential harvests (i.e. between 31 and 38 DAH or between 38 and 45 DAH), there was a significant relationship between the decline in MC and the improvement in *p*_50_ as a proportion of the *p*_50_ of the less mature seed lot (Fig. 2A). Conversely, if the seeds gained moisture between sequential harvests, the greater the amount of water absorbed the greater the potential decline in *p*_50_ (Fig. 2B). The greatest proportional decline in *p*_50_ was by 66 %, from 8.34 to 2.82 d between 38 and 45 DAH, for accession IRGC 125739.

**Fig. 3.**
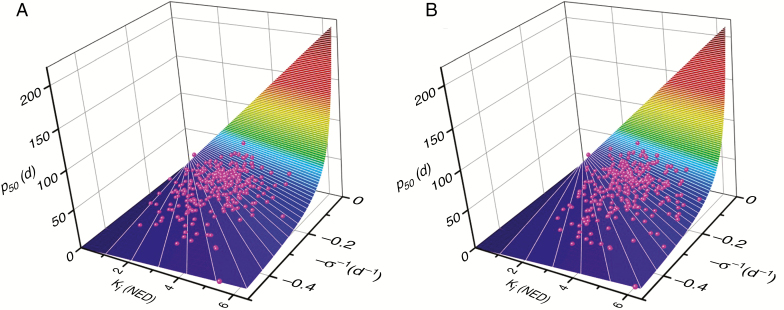
Three-dimensional plots showing the relationship between *K*_i_, –*σ*^−1^ and *p*_50_ (coloured surface) and the estimates of these parameters for seeds of 299 Indica rice accessions (A) harvested at 38 days after heading (DAH) or (B) with the highest estimate of *p*_50_ (harvested at 38 or 45 DAH). Seeds were stored at 45 °C and 10.77–11.66 % moisture content.

Considering seeds harvested at 38 DAH, *K*_i_ [hereafter expressed as *K*_i(38DAH)_] ranged between 0.12 (IRGC 127432) and 5.84 NED (IRGC 127789) with a mean of 2.70 NED (Fig. 3; Table 1). As the initial quality of most seed lots improved at 45 DAH, the range increased overall from 0.53 to 6.29 NED with a mean of 3.32 NED. The slope of the survival curves, −*σ*_(38DAH)_^−1^, ranged between −0.03 and −0.50 d^−1^ with a mean of −0.16 d^−1^. Thus, *σ*_(38DAH)_ ranged between 2 and 33.3 d. Variation in *K*_i(38DAH)_ and *σ*_(38DAH)_ resulted in variation in *p*_50(38DAH)_ between 1.03 and 59.12 d (accessions IRGC 127432 and IRGC 125713, respectively; Supplementary Data Fig. S1B, G). For seeds with the greatest *p*_50_ within an accession, *p*_50(max)_ across the accessions ranged between 5.41 and 59.12 d (accessions IRGC 127410 and IRGC 125713, respectively; Supplementary Data Fig. S1B, F).

**Fig. 4.**
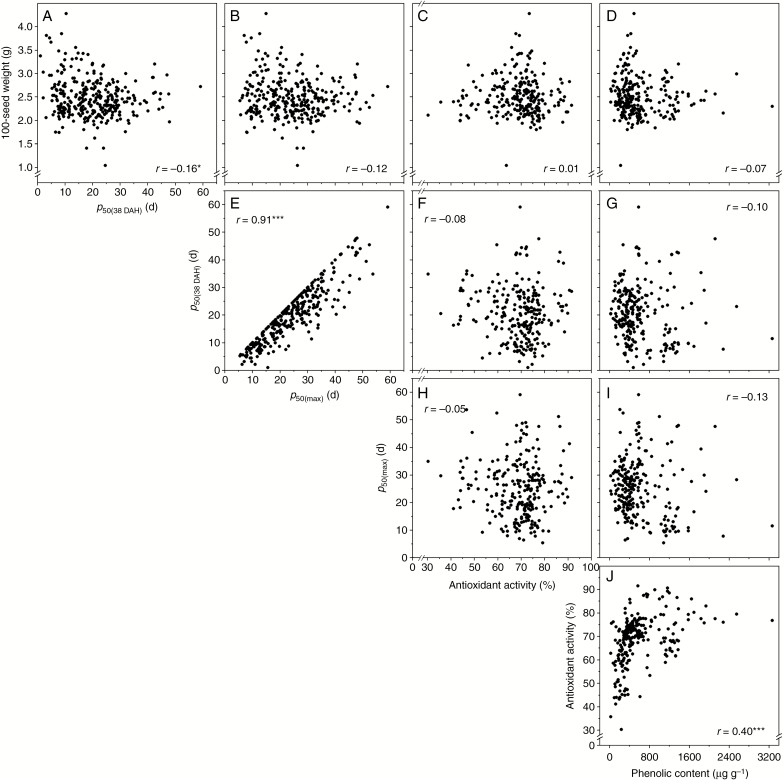
Correlations between 100-seed weight, *p*_50(38DAH)_, *p*_50(max)_, caryopsis antioxidant activity and caryopsis phenolic content for 299 Indica rice accessions. Correlation coefficients (*r*) were significant at *P < 0.05 or ***P < 0.001 (two-sided tests).

The free radical scavenging capacity of caryopsis extracts ranged from 30.30 to 91.50 % (Table 1). Total phenolic compounds (non-enzymatic antioxidants) varied between 18.60 and 2282.70 μg g^−1^ dry weight. There was significant correlation between antioxidant activity and phenolic content (*P* < 0.001; Fig. 4J), but antioxidant activity and phenolic content were not significantly correlated with seed longevity (*p*_50_) for seeds harvested at 38 DAH or with the highest *p*_50_ (Fig. 4F–I). The 100-seed weight was significantly correlated with *p*_50_ for seeds harvested at 38 DAH (*P* < 0.05; Fig. 4A) but not for seeds with the highest *p*_50_ (Fig. 4B), even though there was a significant correlation between the two sets of *p*_50_ values (*P* < 0.001; Fig. 4E).

### GWA analysis

Association analysis was conducted using filtered (MAF > 0.05) 988 k SNP markers. An MLM detected several loci that were associated with individual traits (Supplementary Data Fig. S2), and a number of co-localized trait associations. For example, a haplotype containing marker 10706067855 at position 6 067 855 on chromosome 7 was strongly associated with antioxidant capacity (*P =* 2.82E−11) and total phenolic compounds (*P* = 4.98E−17). However, seed longevity loci did not overlap with any other loci detected for other traits in this study.

**Fig. 5.**
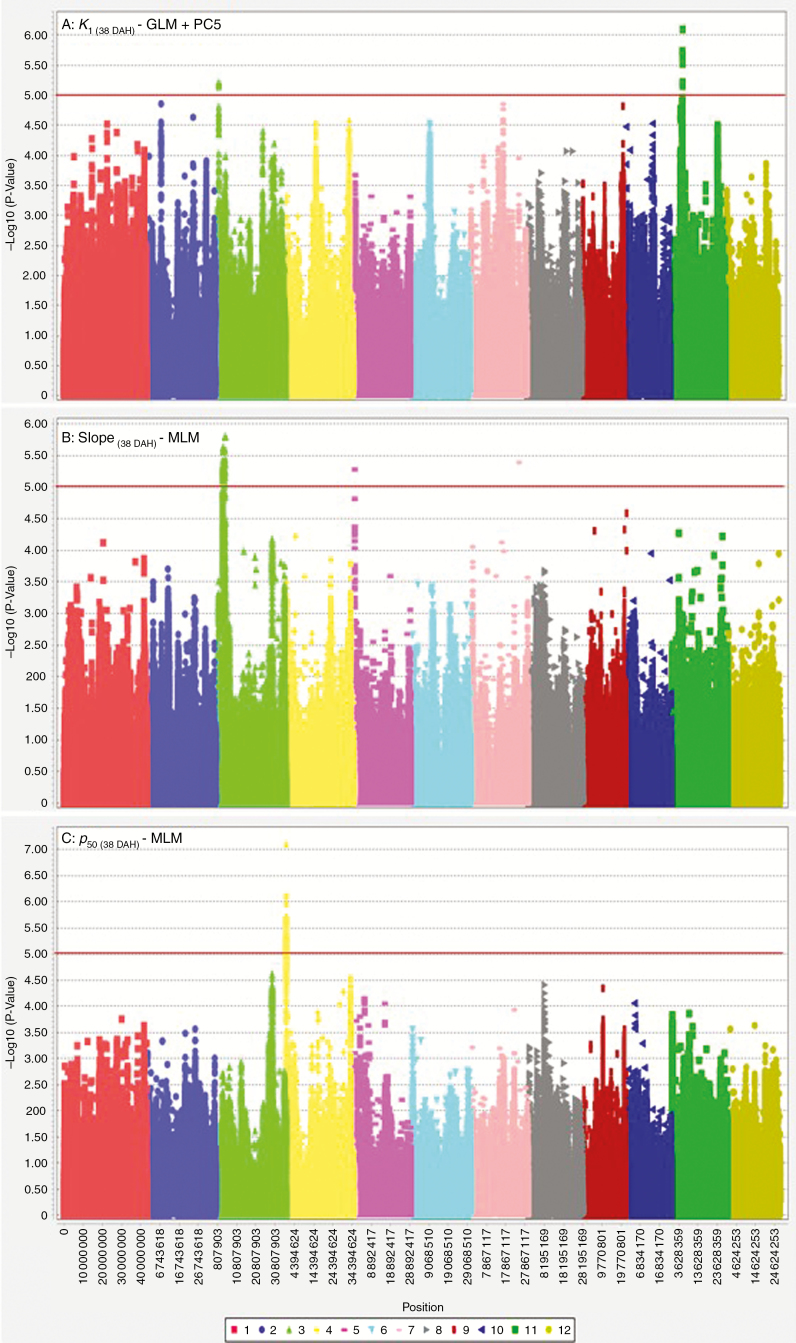
GWA analysis of *K*_i_, –*σ*^−1^ (slope) and *p*_50_ for seeds harvested at 38 days after heading (DAH). Harvest moisture content was included as a covariate of *K*_i_ and *p*_50_. Plots above the red threshold line are significantly associated with traits. Different colours refer to the 12 different chromosomes (see box).

**Fig. 6.**
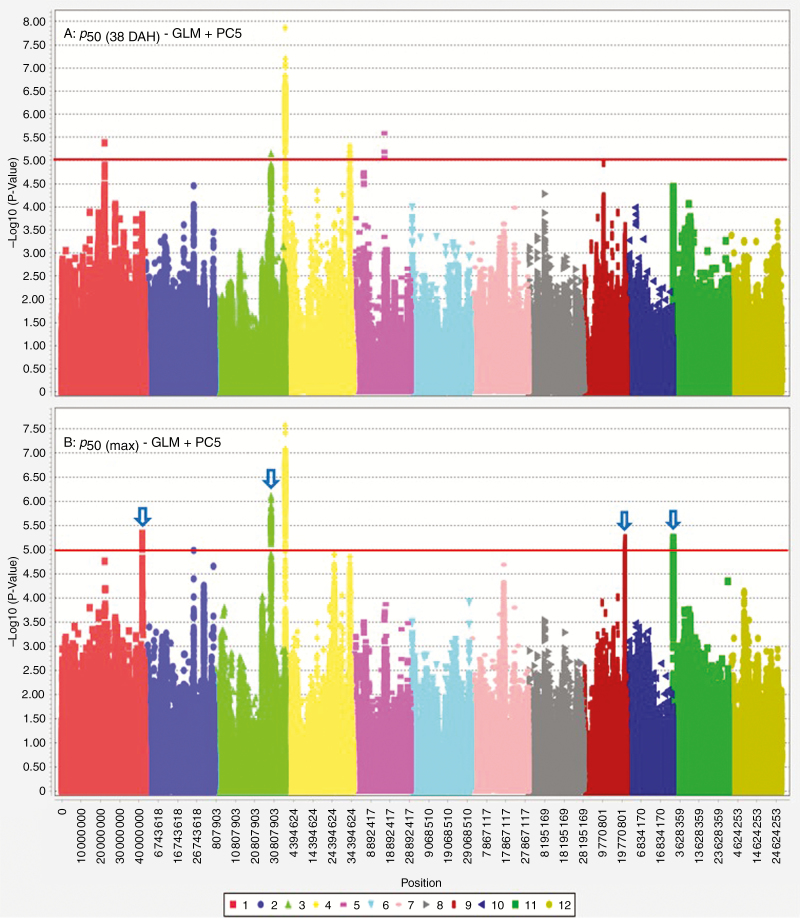
GWA analysis of *p*_50_ of Indica rice accessions harvested at (A) 38 days after heading (DAH) and (B) either 38 or 45 DAH (whichever gave the highest *p*_50_ for each accession). Harvest moisture content was used as a covariate of *p*_50_. Plots above the red threshold line are significantly associated with the trait. Different colours refer to the 12 different chromosomes (see box). Arrows indicate loci that appeared when the maximum *p*_50_ for each accession was used.

**Fig. 7.**
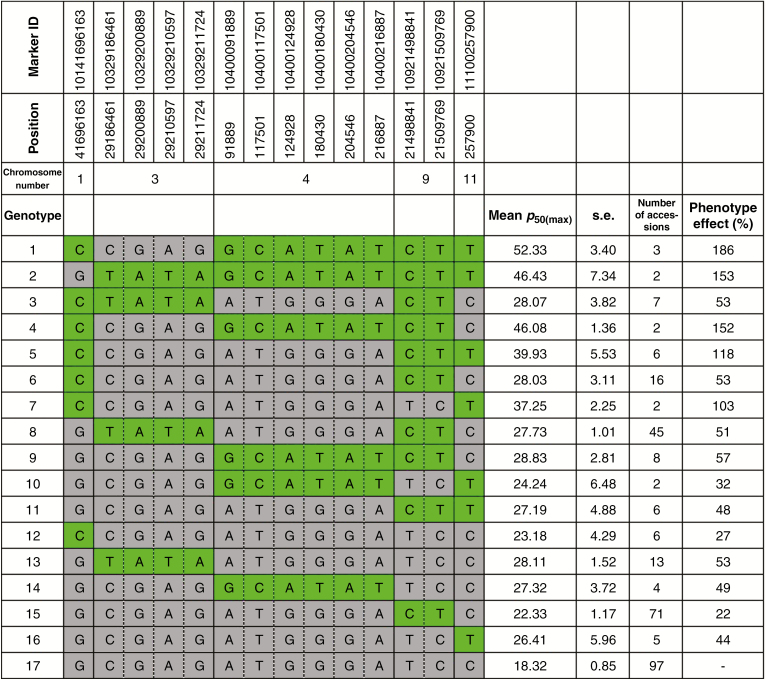
Haplotype effects on seed longevity (*p*_50_) in a diverse Indica rice panel.

Harvest MC was used as a covariate of seed longevity traits. For seeds harvested at 38 DAH (typical harvest time in the International Rice Genebank), a single major peak on chromosome 4 associated with *p*_50(38DAH)_ was consistently detected with both MLM and GLM analyses (Fig. 5). Two peaks associated with *K*_i(38DAH)_, on chromosomes 3 and 11, were detected, but *P* values were below the MLM threshold, likely due to underestimated genetic effects, as indicated by the quantile–quantile plot (Supplementary Data Fig. S2). For the slope [−*σ*_(38DAH)_^−1^], a consistent major peak on chromosome 3 was detected in both MLM and GLM. Further association analysis, to identify additive genetic effects in relation to continued on-plant seed maturation, was performed using the maximum *p*_50_ observed for an accession (i.e. for seeds harvested at either 38 or 45 DAH; Fig. 6). Compared with the Manhattan plot of 38 DAH seed lots, four additional loci appeared on chromosomes 1, 3, 9 and 11. The allelic effects of identified loci were estimated through haplotype mapping (Fig. 7). The presence of favourable haplotypes (shaded green) at one or two loci (genotypes 6–16) was associated with moderate enhancements of seed longevity (*p*_50_), by 22–103 % compared with genotype 17 having unfavourable haplotypes (shaded grey) at all loci. Accessions with favourable haplotypes at three loci (genotypes 3–5) showed 53–152 % increase in longevity, while accessions with four-locus combinations of favourable haplotypes (genotypes 1 and 2) showed enhancements of 153–186 %, suggesting additive effects of the haplotypes.

### Candidate gene selection

Our GWA peaks tended to locate inside linkage disequilibrium blocks (data not shown). The size of each GWA peak was determined based on the physical distance between markers with subsequently increased and decreased *P* values between a threshold. Since we do not know which markers are causal SNPs, we first considered all genes located at GWAS peaks as potentially associated with the trait. Annotation using MSU7 (Kawahara *et al.*, 2013) identified 66 genes located in the eight GWA peak regions (on chromosomes 1, 3, 4, 9 and 11 for *p*_50_; on chromosomes 3 and 11 for *K*_i_; and on chromosome 3 for slope) (Figs 5 and 6; Supplementary Data Table S3). Based on gene annotation plus ontology with networking genes (RiceNet v2, accessed on 2 November 2017; Lee *et al.*, 2015), 36 genes were considered as potentially relevant to seed longevity. We also considered whether gene expression occurred in seed tissues (The Bio-Analytic Resource for Plant Biology Database). Using the SNP-Seek Database (Mansueto *et al.*, 2017), we aligned allelic variations and focused on gene regions where significant *P*-value markers were located in exons and/or introns. In total, eight genes were identified as *a priori* candidate genes associated with seed longevity (Table 2).

**Table 2.** List of *a priori* candidate genes enhancing seed longevity of 299 Indica rice accessions

No	Controlled trait	Candidate gene ID	Chromosome	Position	Annotation	*P* value of most significant marker^1^	Allele^2^	Allele effect (%)	Network gene^3^	Network gene ontology^3^
1	*p*_50_ (+desicc.)	LOC_Os03g51050	3	29185006-29190361	Peptide transporter PTR2	4.38E−06/ 6.50E−05	T/C (22)	24.6	LOC_Os03g45170/ LOC_Os10g30090/ LOC_Os03g54000	Amino acid transmembrane transport/”/oligopeptide transport
2		LOC_Os04g01160	4	121631–125374	Zinc finger family protein	3.23E−07/ 1.06E−05	A/G (7)	49.5	LOC_Os06g04560/ LOC_Os07g14540/	Root development/photoperiodism; flowering
3		LOC_Os04g01280	4	215929–219439	Glycosyltransferase family 43 protein	3.86E−08/ 1.06E−05	G/T (8)	51.8	LOC_Os03g16980/ LOC_Os12g25700/ LOC_Os12g25690	D-xylose metabolic process/UDP-glucuronate biosynthetic process; oxidation reduction/”
4		LOC_Os09g37250	9	21515853-21517621	High mobility group	5.78E−06/ 3.02E−05	T/C (56)	29.1	LOC_Os07g44690/ LOC_Os04g40420/ LOC_Os02g10060	Regulation of transcription, DNA-dependent/regulation of gene expression/”
5		LOC_Os11g01439	11	256827–262011	Chloroplast unusual positioning protein	6.37E−06/ 8.08E−05	C/T (10)	49.0	LOC_Os06g09880/ LOC_Os02g01150/ LOC_Os08g45190	Maltose metabolic process; embryonic development/ oxidation reduction/response to sucrose stimulus
6	Slope	LOC_Os03g06890	3	3483353-3485708	DUF593 domain containing protein	9.47E−06/ 2.63E−06	C/T (91)	42.1	LOC_Os09g32440/ LOC_Os03g11140/ LOC_Os02g49070	Response to ABA; root development/positive regulation of GTPase activity/”
7		LOC_Os03g06900	3	3489869-3500130	DNA topoisomerase 3 protein	1.01E−05/ 2.07E−06	G/C (89)	42.1	LOC_Os02g53680/ LOC_Os09g24220/ LOC_Os04g54340	Double-strand break repair/mismatch repair/”
8	*K*_i_	LOC_Os03g03870	3	1752160-1758218	DNA-binding bromodomain-containing protein	6.42E−06/ 2.70E−02	G/A (15)	25.7	LOC_Os01g65900/ LOC_Os06g03990/ LOC_Os10g28040	Transcription, DNA-dependent/cellular amino acid metabolic process/regulation of transcription

Double quotation marks mean same gene ontology as front gene has.

^1^ *P*-values of GLM PC_5_/MLM of SNP marker within candidate gene region.

^2^Favourable/unfavourable alleles; numbers in parentheses indicate the frequency (%) of favourable alleles; shaded and non-shaded alleles locate in exons and introns, respectively.

^3^Direct neighbourhood with high log likelihood scores (top 3) and ontology potentially relevant to seed longevity.

## DISCUSSION

Understanding the molecular basis of seed longevity and why seeds of some species have the potential to remain alive for decades or centuries under optimal storage conditions is an ongoing focus of seed biology. It is not only of direct relevance to those concerned with storing seeds for long periods of time, such as genebank managers, but also, since seed longevity is an inherent aspect of seed physiological quality, to anyone involved in producing or using seeds. Seeds with greater longevity will retain their planting value for longer periods, regardless of storage conditions. There is considerable variation among orthodox species in seed longevity (Priestley *et al.*, 1985; Hong *et al.*, 1996); thus, it has been possible to identify trends in relative seed longevity associated with, for example, the climate in the country of origin (Walters *et al.*, 2005; Probert *et al.*, 2009). There is much less variation within a species or even a genus. Early studies assumed that the ‘rate’ of loss of viability, i.e. the slope of the survival curve (−*σ*^−1^) did not differ between seed lots of the same species stored at the same MC and temperature (Ellis and Roberts, 1980). Thus, it was suggested that only the initial quality of the seeds, through *K*_i_, determines the longevity of a seed lot of a particular species in storage. However, Whitehouse *et al.* (2018) have found that both *K*_i_ and *σ* can vary among rice varieties. Variation in *σ* has also been reported for foxglove (*Digitalis purpurea*; Hay *et al.*, 1997) and *Trifolium ambiguum* (Hay *et al.*, 2010) seeds. Similarly, here we found that there was considerable variation in both *K*_i_, as expected (between 0.53 and 6.29 NED for seeds with maximum longevity), and *σ* (between 2 and 33.3 d) within 299 Indica rice varieties (Table 1). This is the first time variation in both seed longevity parameters have been explored so extensively through seed storage experiments (Hay *et al.*, 2019). Given this variation, we have been able to conduct GWA analysis for the different longevity parameters (*K*_i_, −*σ*^−1^ and *p*_50_).

Seed longevity is known to improve throughout seed development, as the seeds acquire desiccation tolerance and during the first part of the desiccation phase, when the seeds are still metabolically active but starting to dry to equilibrium with ambient conditions (Ellis and Pieta Filho, 1992; Angelovici *et al.*, 2010; Hay *et al.*, 2010; Chatelain *et al.*, 2012; Whitehouse *et al.*, 2015). This late ‘accumulation’ of seed longevity can make it difficult, especially for a shatter-resistant crop like rice, to know when to harvest seeds for maximum physiological quality. Previous studies (Kameswara Rao and Jackson, 1996*a*, *b*) have identified the optimum harvest time for rice accessions to be at around 35 d after anthesis (DAA, approximately equivalent to DAH − 1). More recently, Whitehouse *et al.* (2015) found that the harvest date and hence MC at harvest was more critical than chronological age. For the study reported here, we attempted to take into account the potential effect of seed maturity by harvesting each accession on three occasions and characterizing the seed survival curves for each seed lot. The mean (across all accessions) 100-seed weight changed significantly, but only slightly between 31 and 38 DAH (mean ± s.d. change +0.017 ± 0.103 g) and between 38 and 45 DAH (mean ± s.d. change −0.012 ± 0.097 g) (Fig. 1C, D), confirming that the seeds were harvested after mass maturity. Indeed, Kameswara Rao and Jackson (1996*b*) found that for rice accessions grown in the same location and season, mass maturity occurs between 18.5 and 21.6 DAA. Hence, we infer that the rice seeds in our study were in the desiccation phase of seed development. Of the 299 accessions included in the study, 250 declined in MC between 31 and 38 DAH, of which 139 continued to lose moisture between 38 and 45 DAH. However, harvest MC depends on ambient conditions and, as seen in Whitehouse *et al.* (2015), the general trend was for harvest MC to decline over the harvesting period (Fig. 1A). Thus, seeds that were harvested later were less likely to show continued decline in MC over successive harvests, because they had already dried by 31 DAH.

Seed longevity was influenced by the harvest MC. For seeds that were drying on the plant, there was, on average, an 8.7 % improvement in longevity (*p*_50_) for every 1 % reduction in MC (Fig. 2A). Conversely, if seeds took up water between consecutive harvests, *p*_50_ was likely to decline (Fig. 2B). A decline in seed longevity if harvest is delayed is not unexpected (Kameswara Rao and Jackson, 1996*a*, *b*; Hay *et al.*, 2015; Nasehzadeh and Ellis, 2017). In the case of non-shattering wild species, it has been suggested that determining the moisture status of a sample of seeds can be used to determine whether the seeds are ready to harvest and/or the optimum way to dry the seeds (or not) after harvest (Hay and Probert, 2011). For rice, it has been found that if the seeds are harvested moist (>16.48 % MC), the subsequent longevity in air-dry storage is improved if the seeds are dried at a relatively high temperature (45 °C; Whitehouse *et al.*, 2018). Thus, high-temperature drying substitutes for on-plant desiccation, and even stimulates an enhanced response (Whitehouse *et al.*, 2017), when ambient conditions are too humid to allow drying *in situ*. In the present study, all seeds were dried at 15 % RH/15 °C, conditions that conform with the Genebank Standards for seed drying (FAO, 2014). The results of the GWA analysis might have differed if the seeds had been initially dried at the higher temperature; this is now standard practice for regenerated accessions at the International Rice Genebank.

Nagel *et al.* (2015) found that antioxidants in barley seeds were highly associated with longevity. In our study, *p*_50_ was not correlated with free radical scavenging capacity of antioxidants in the caryopsis or total phenolics (Fig. 4E–I). This could be partly due to our assay, which measured all antioxidants rather than specific antioxidants. Our recent study on correlation between seed longevity and vitamin E in the caryopses of 20 rice accessions found that among eight types of vitamin E, only γ-tocotrienol was significantly correlated with seed longevity (Lee *et al.*, 2017). Another possibility is that antioxidants play a lesser role in the seed longevity of Indica rice. Seed longevity varies across subgroups of rice (Lee *et al.*, 2017). This might be due to intraspecific variation in genomic structure. Hence, each subgroup might have different mechanism(s) conferring extension of seed longevity. Candidate gene network ontology identified through the current study also suggests that oxidation reduction may play an important role in seed longevity (Table 2). For example, LOC_Os12g25690 and LOC_Os12g25700, part of the network of candidate gene LOC_Os04g01280, are related to oxidation reduction. Therefore, individual antioxidants need to be tested further for association with seed longevity. Ideally, this would involve looking at embryo-specific antioxidant levels rather than whole seeds.

Based on the database of network prioritization server (RiceNet v2), various genes in networks that are connected to our candidate genes were identified. We prioritized direct neighbourhoods of candidate genes, which, through the combination of log likelihood scores (top 3), annotation and ontology, were considered relevant to seed longevity mechanisms (Table 2, Supplementary Data Table S3). Sasaki *et al.* (2015) reported that ‘*TPP7’* and/or its flanking gene on chromosome 9 conferred the difference in seed longevity between seeds of ‘Milyang23’ (an Indica variety, high longevity) and ‘Akihikari’ (a temperate Japonica variety, poor longevity). However, these genes were not indicated as important in the Indica panel used in this study. Indeed, a large deletion on ‘*TPP7’* is present in many Indica accessions (Kretzschmar *et al.*, 2015).

Association analysis was conducted for *K*_i_, −*σ*^−1^ and *p*_50_; loci for each trait were identified in different locations (Supplementary Data Fig. S2). Ontology-mediated gene networks suggested a common mechanism of seed longevity across longevity parameters: DNA-dependent transcription and repair of damaged DNA (Table 2). Waterworth *et al.* (2016) showed how sensor kinases control DNA repair and the germination of aged *Arabidopsis* seeds. A similar mechanism was reported for *Medicago truncatula* seeds (Verdier *et al.*, 2013). In their results, unfolded protein binding and RNA processing genes were highly associated with seed longevity. LOC_Os04g01160 (SOR1) (Hanzawa *et al.*, 2013) and LOC_Os11g01439 (RLCK57) (Vij *et al.*, 2008), associated with *p*_50_, and LOC_Os03g06890, associated with slope, were annotated to root and embryonic development. LOC_Os03g51050 (PTR8) (Léran *et al.*, 2014), LOC_Os09g37250 and LOC_Os11g01439 (RLCK57) were ‘additionally associated’ with on-plant desiccation due to delayed harvest (Fig. 6). LOC_Os04g01280 and LOC_Os11g01439 (RLCK57) with the largest allelic effects on *p*_50_ (51.8 and 49.0 %, respectively) are involved in sugar metabolism and reactive oxygen species scavenging (Bailly, 2004; ElSayed *et al.*, 2014). Both mechanisms were previously demonstrated as determinants of seed longevity. LOC_Os04g01280 and LOC_Os04g01160 are known to control auxin induced root gravitropism and root architecture, such as primary root length and lateral root number (Shi *et al.*, 2009). As the main advantage of GWAS using a diverse rice panel with high-density genotype data, we provide the first report of these genes as potentially being involved in seed longevity. Similarly, Righetti *et al.* (2015) revealed that pathogen-resistant genes indeed play an important role in seed longevity extension.

From a genebank management perspective, testing Indica accessions for favourable haplotypes with respect to longevity through cost-efficient genotyping assays will make it possible to flag particular seed lots requiring less frequent cycles of seed multiplication. Conversely, Indica accessions without favourable haplotypes should be prioritized for more frequent viability monitoring to ensure that seed lots are above the viability standard. Seed longevity markers are also important from a breeding perspective. Rapid decline in seed viability leaves farmers vulnerable in terms of seed security, especially since the incidence of hot, humid weather is likely to increase under climate change. Viable seeds are critical to successful crop establishment and weed competitiveness during the early stages of crop growth, particularly under direct-seeded conditions, and thus improved seed longevity will be an increasingly important adaptive trait for farmers. In the context of direct seeding of rice, longevity should be recognized as part of the essential trait package. It could be of particular interest to hybrid programmes, where the value of the seeds is higher and poor germination becomes a liability. The loci detected in this study demonstrated sufficient improvement in longevity to merit further validation for potential use in breeding programmes for direct-seeded and hybrid varieties.

For this study, as well as characterizing the seed survival curve and taking into account the effect of seed maturity, the seed MC used was lower than in many other studies involving ‘artificial ageing’ or ‘controlled deterioration’ (Clerkx *et al.*, 2004; Xue *et al.*, 2008; Chatelain *et al.*, 2012; Rehman Arif *et al.*, 2012; Li *et al.*, 2014; Hay *et al.*, 2019). Some studies have additionally considered storage under ‘natural’ or ‘conventional’ storage conditions, at lower MC, and identified different QTLs contributing to differences in longevity in the different environments (Schwember and Bradford, 2010; Nagel *et al.*, 2011). The target MC for the storage experiments here was 10.9 %, the predicted MC of desorbing rice seeds at 60 % RH. These conditions were used to ensure the results were comparable with other studies on cultivated and wild rice (Hay *et al.*, 2015; Whitehouse *et al*., 2015, 2017; Timple and Hay, 2018), and across diverse species (Probert *et al.*, 2009; Mondoni *et al.*, 2011; Merritt *et al.*, 2014). Interestingly, this MC is less than that recommended for farmers and seed companies storing rice seeds (12–14 %). It is also within the linear part of the moisture desorption isotherm, where we expect there to be a linear relationship between seed longevity (log_10_*σ*) and MC (log_10_MC; Hay and Timple, 2016). In the case of accelerated ageing or controlled deterioration experiments that are carried out at much higher MC, particularly those above 85 % RH, different metabolic process might be expected to be occurring (Vertucci and Leopold, 1984), and hence identification of different ‘seed longevity’ loci is probable. Since we used a lower RH, we expect that seed lots (accessions) that lose viability more quickly in this storage environment would also lose viability more quickly in genebank storage. The accessions used are relatively new, derived from single-seed descent of original accessions, and hence there is not yet enough viability monitoring data to confirm that the identified genes may be influencing longevity in long-term storage. Nonetheless, based on the results of the GWA, we propose the use of the candidate markers to predict the relative longevity of Indica rice accessions to better manage the International Rice Genebank collection.

## SUPPLEMENTARY DATA

Supplementary data are available online at https://academic.oup.com/aob and consist of the following. Table S1: origin of a diverse Indica rice panel used in the GWAS of seed longevity. Table S2: information on 299 Indica rice accessions used in the GWAS of seed longevity. Table S3: list of candidate genes associated with seed longevity traits of 299 Indica rice accessions. Figure S1: seed survival curves of 299 Indica rice accessions. Figure S2: GWA analysis on traits of 299 Indica rice accessions measured in this study. File S1: additional references for information on 299 Indica rice accessions.

mcz093_suppl_Supplementary_Figure_S1

mcz093_suppl_Supplementary_Figure_S2

mcz093_suppl_Supplementary_Table_S1

mcz093_suppl_Supplementary_Table_S2

mcz093_suppl_Supplementary_Table_S3

## FUNDING

This research was funded through the CGIAR Research Program for Managing and Sustaining Crop Collections.
