# Supplementary material for: Variation in seed longevity among diverse Indica rice varieties
Source: Ann Bot. 2019 Jun 10;124(3):447–60. doi: 10.1093/aob/mcz093 (PMC6798842; doi:10.1093/aob/mcz093)
Supplement: mcz093_suppl_Supplementary_Figure_S1 [file mcz093_suppl_supplementary_figure_s1.pptx]

## Slide 1
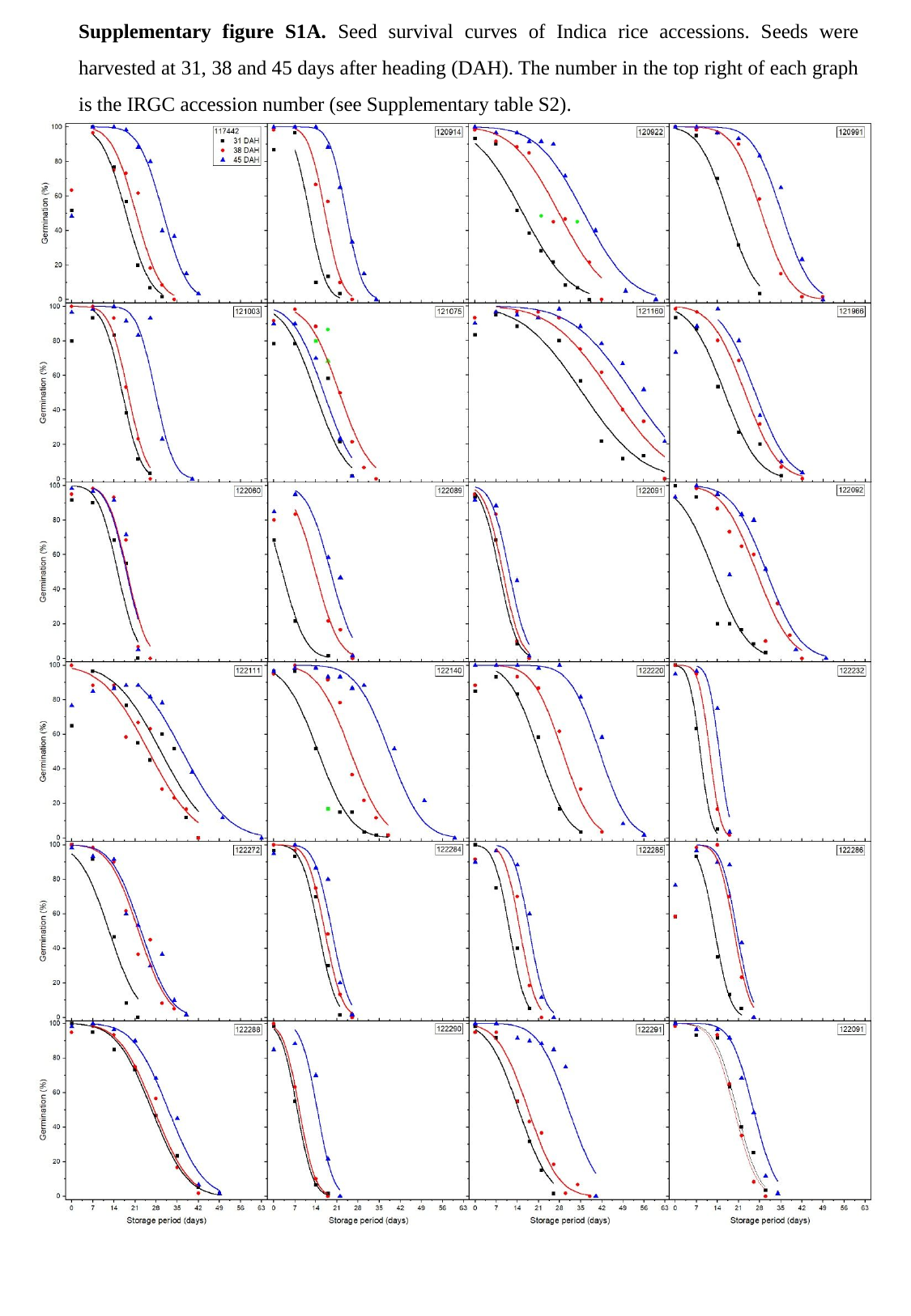

Supplementary figure S1A. Seed survival curves of Indica rice accessions. Seeds were harvested at 31, 38 and 45 days after heading (DAH). The number in the top right of each graph is the IRGC accession number (see Supplementary table S2).

## Slide 2
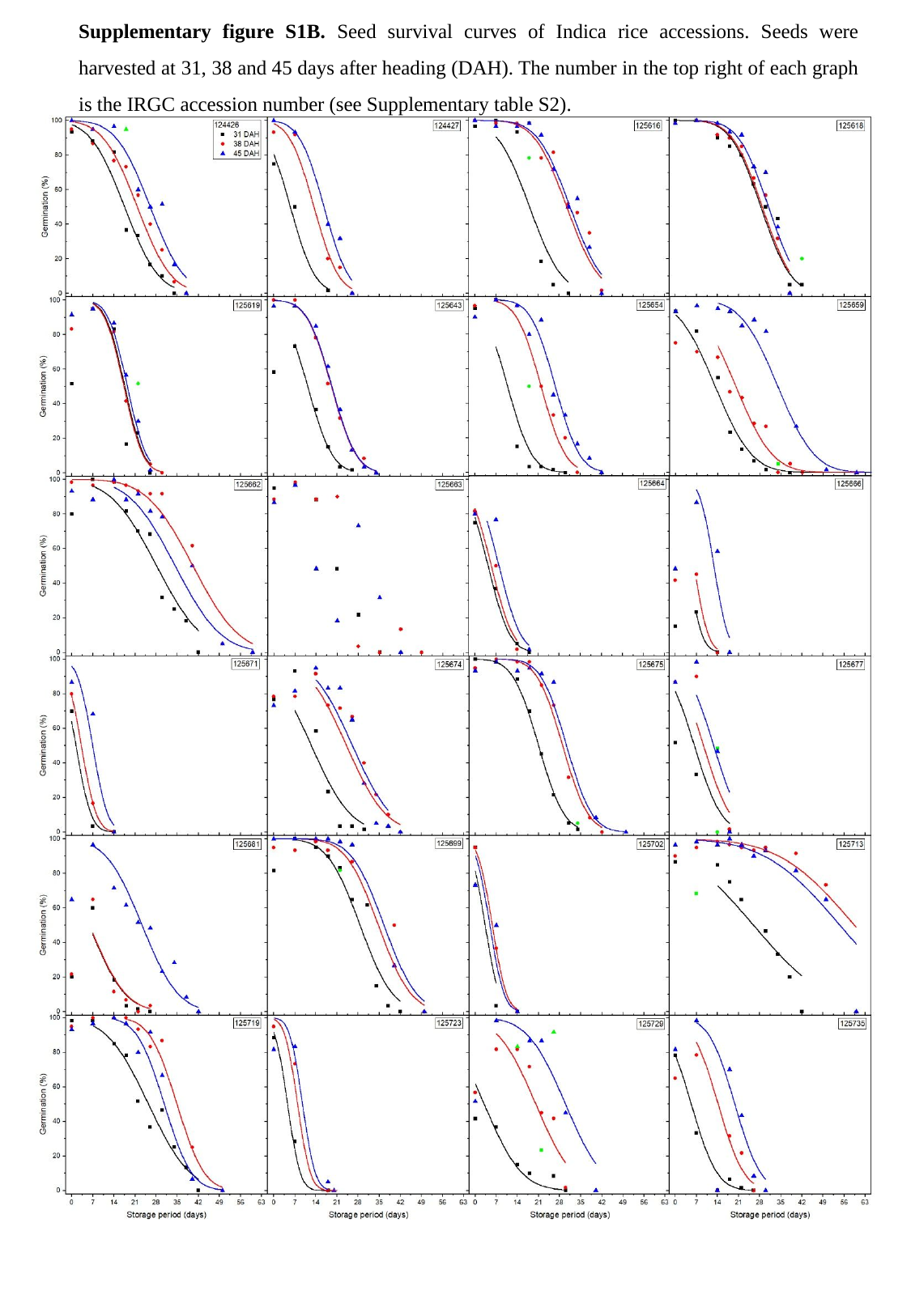

Supplementary figure S1B. Seed survival curves of Indica rice accessions. Seeds were harvested at 31, 38 and 45 days after heading (DAH). The number in the top right of each graph is the IRGC accession number (see Supplementary table S2).

## Slide 3
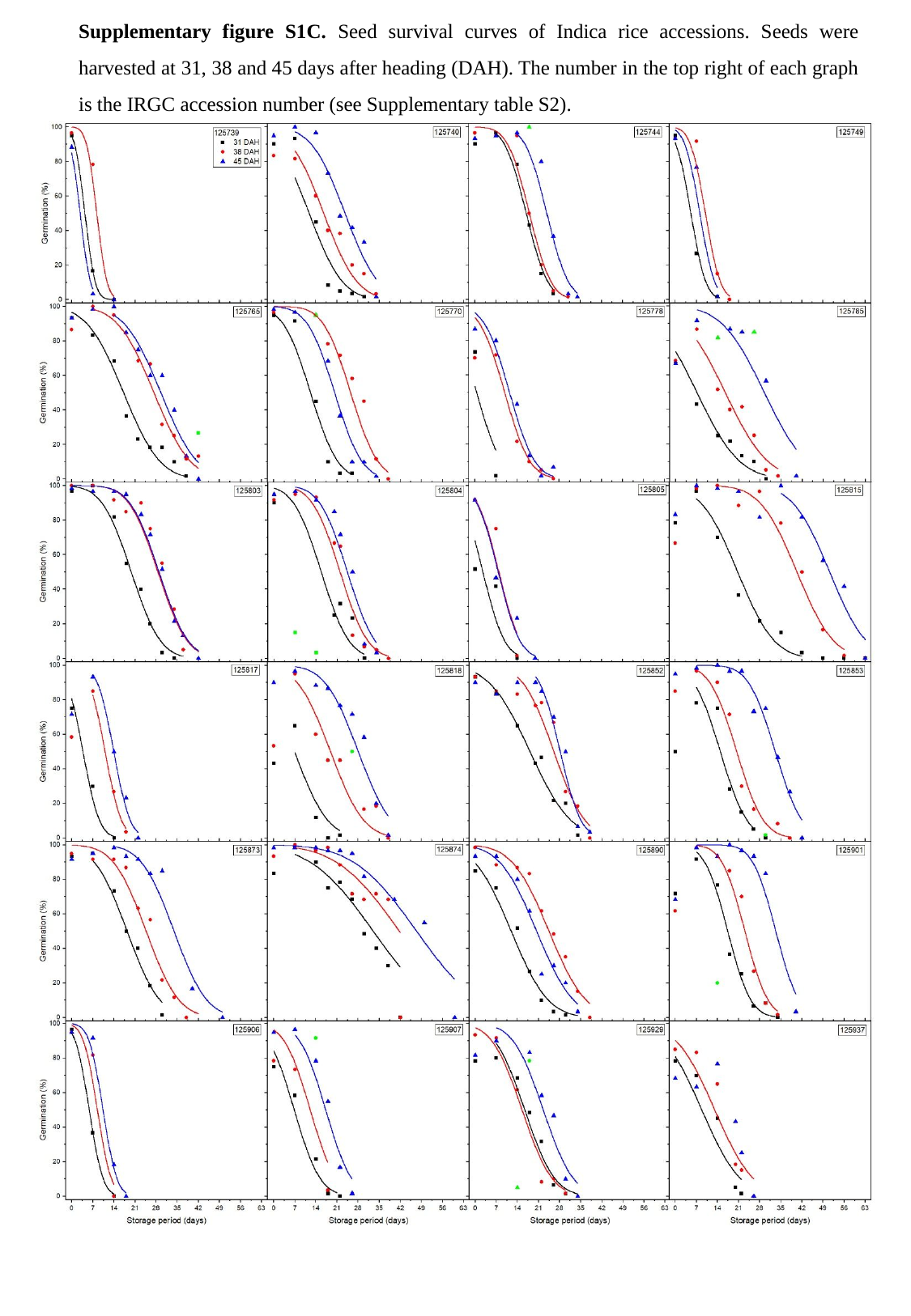

Supplementary figure S1C. Seed survival curves of Indica rice accessions. Seeds were harvested at 31, 38 and 45 days after heading (DAH). The number in the top right of each graph is the IRGC accession number (see Supplementary table S2).

## Slide 4
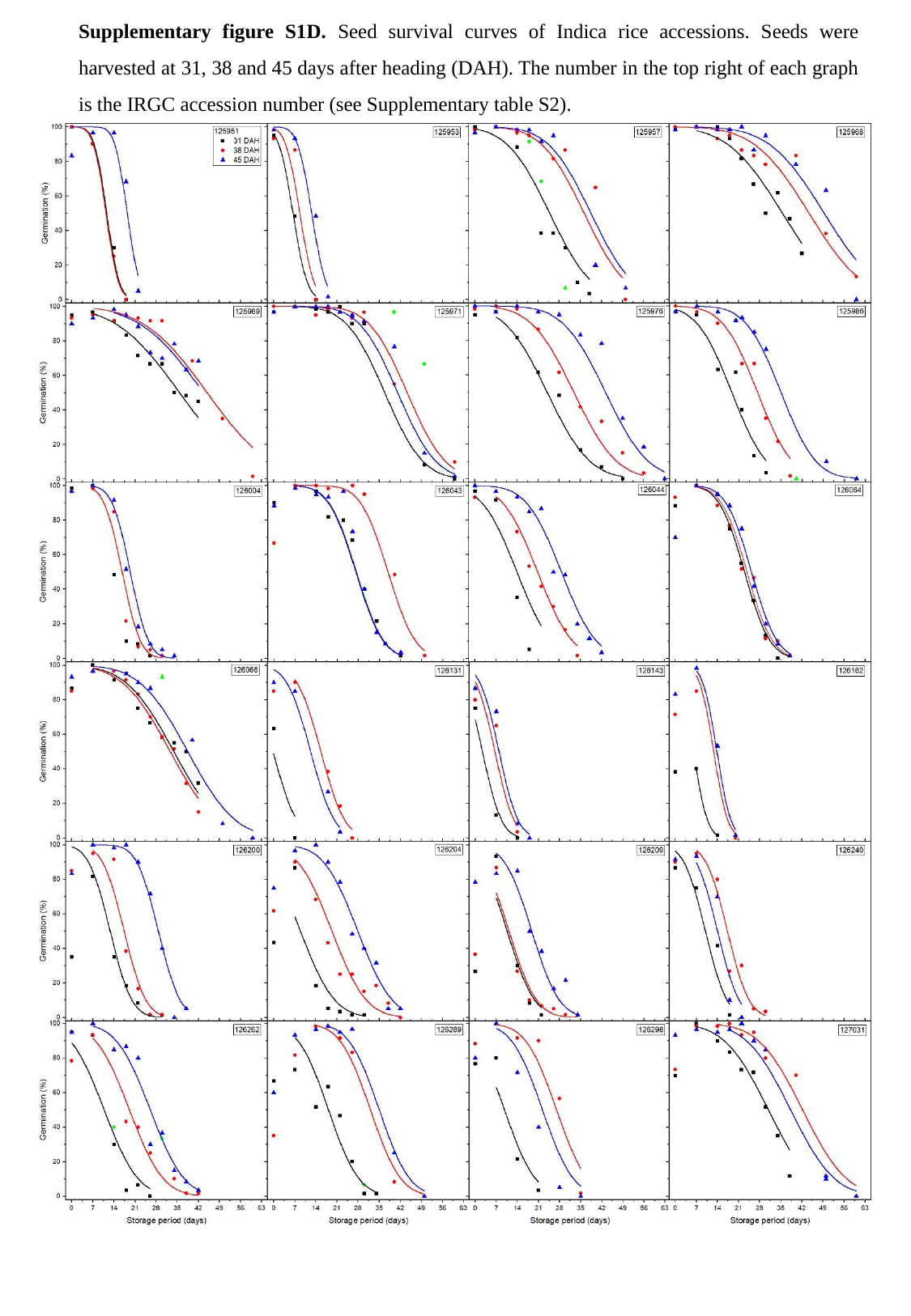

Supplementary figure S1D. Seed survival curves of Indica rice accessions. Seeds were harvested at 31, 38 and 45 days after heading (DAH). The number in the top right of each graph is the IRGC accession number (see Supplementary table S2).

## Slide 5
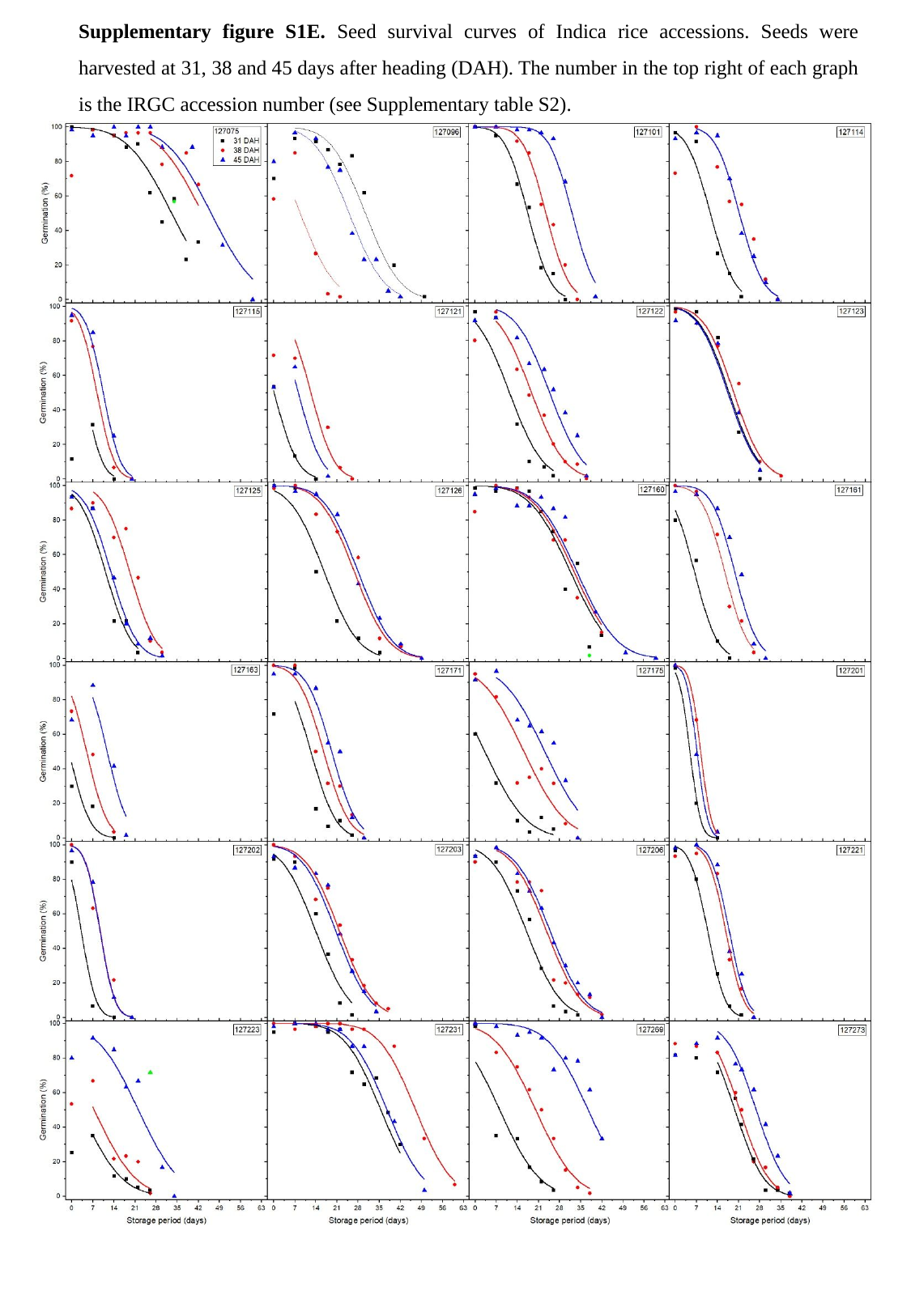

Supplementary figure S1E. Seed survival curves of Indica rice accessions. Seeds were harvested at 31, 38 and 45 days after heading (DAH). The number in the top right of each graph is the IRGC accession number (see Supplementary table S2).

## Slide 6
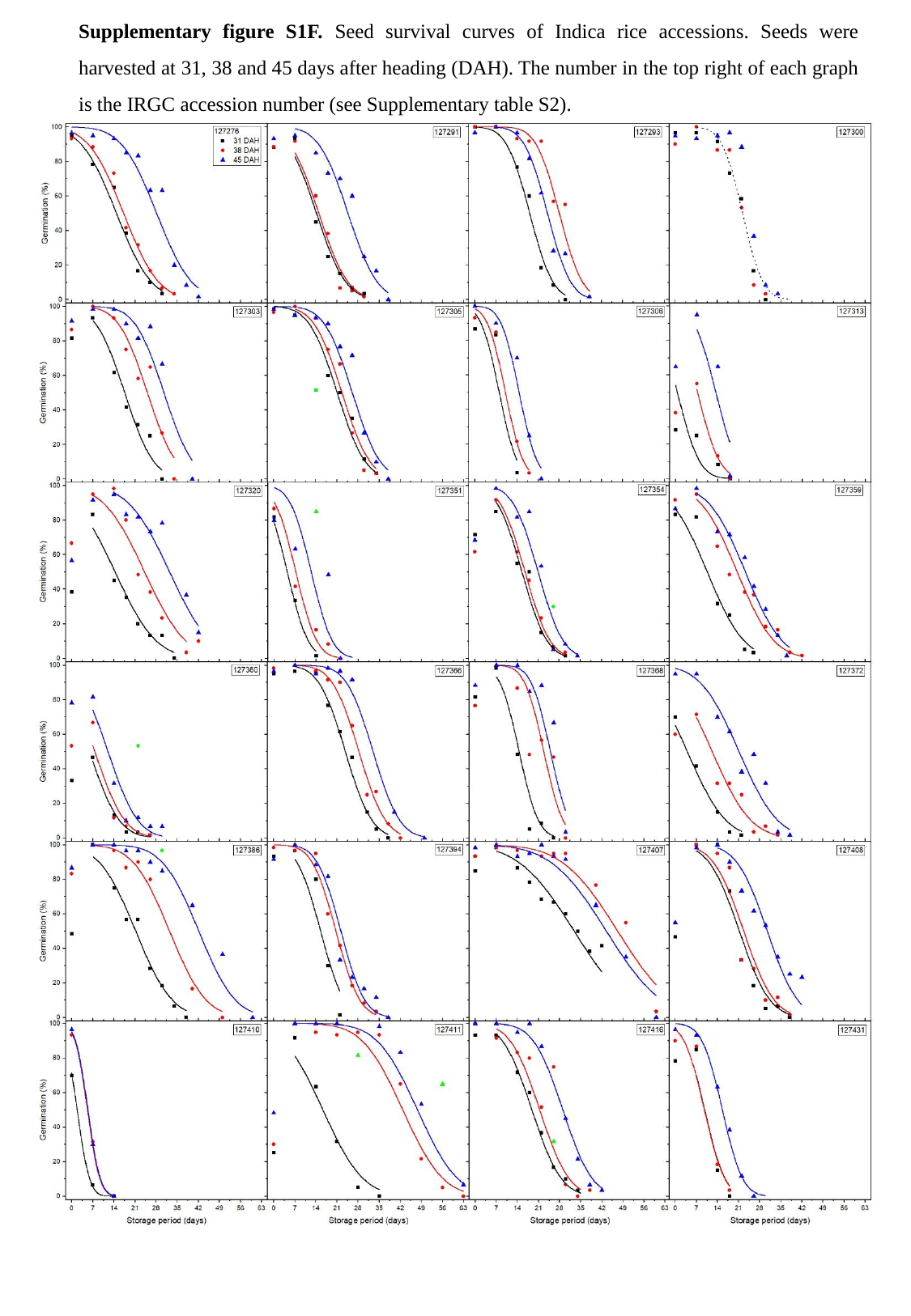

Supplementary figure S1F. Seed survival curves of Indica rice accessions. Seeds were harvested at 31, 38 and 45 days after heading (DAH). The number in the top right of each graph is the IRGC accession number (see Supplementary table S2).

## Slide 7
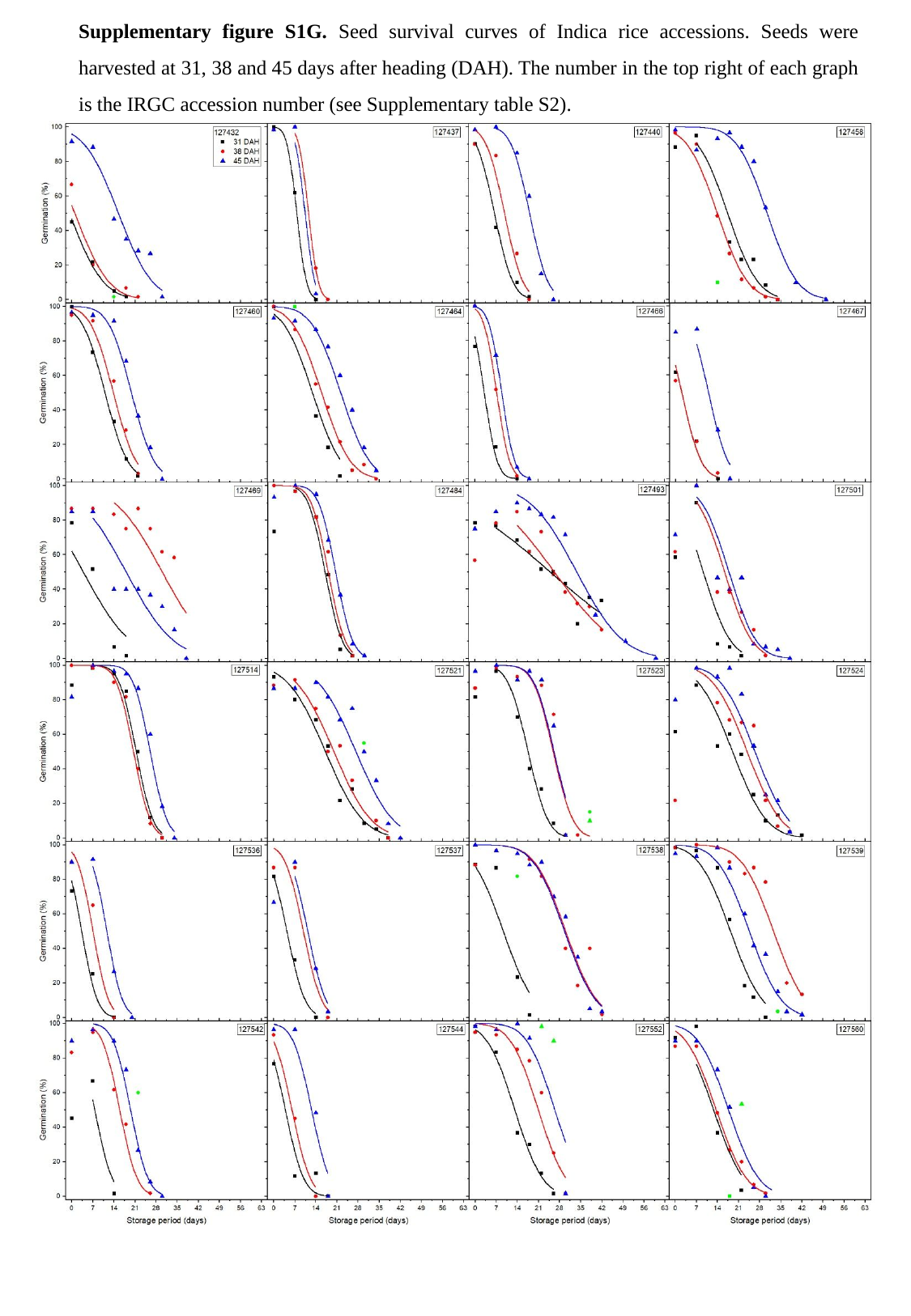

Supplementary figure S1G. Seed survival curves of Indica rice accessions. Seeds were harvested at 31, 38 and 45 days after heading (DAH). The number in the top right of each graph is the IRGC accession number (see Supplementary table S2).

## Slide 8
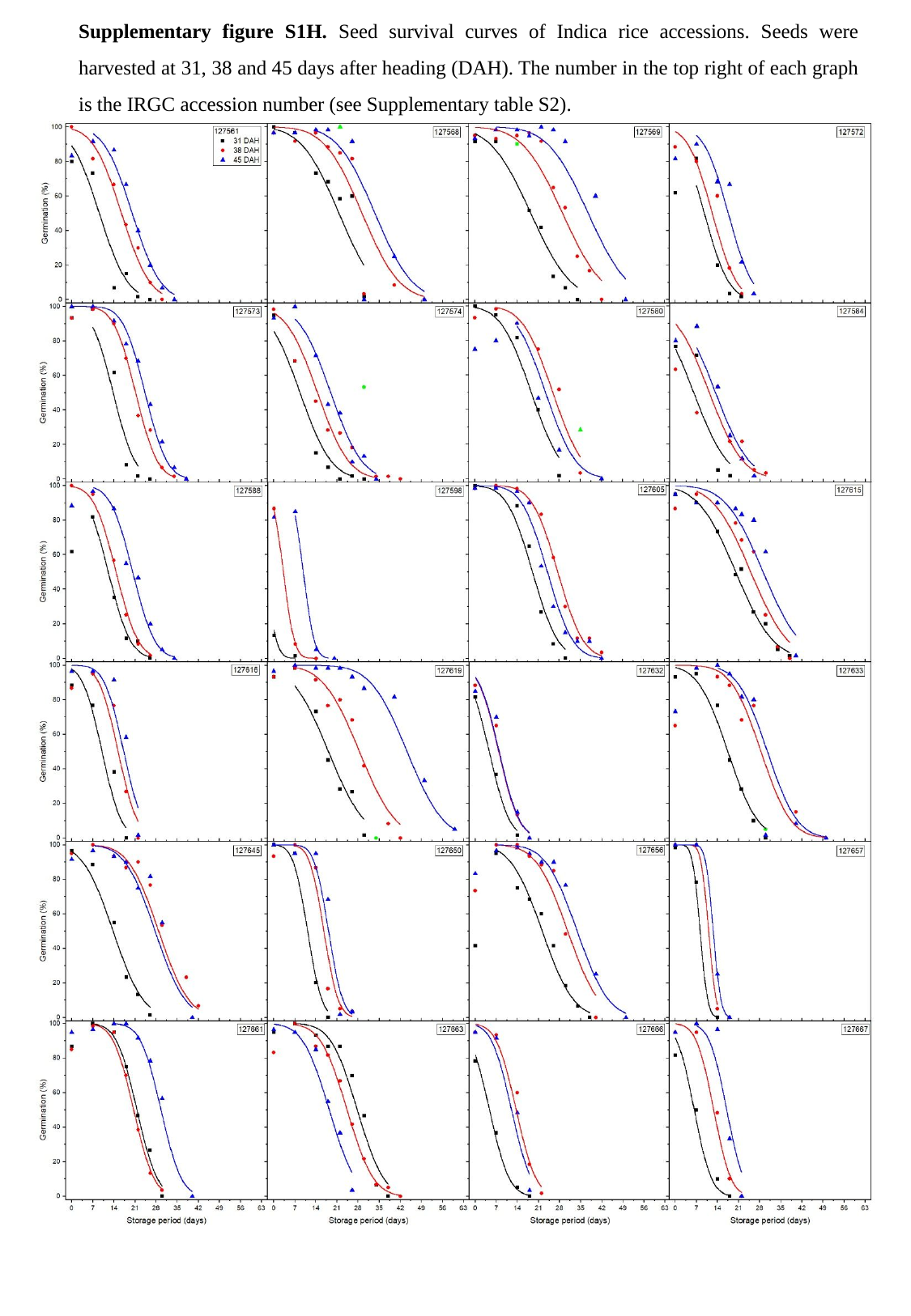

Supplementary figure S1H. Seed survival curves of Indica rice accessions. Seeds were harvested at 31, 38 and 45 days after heading (DAH). The number in the top right of each graph is the IRGC accession number (see Supplementary table S2).

## Slide 9
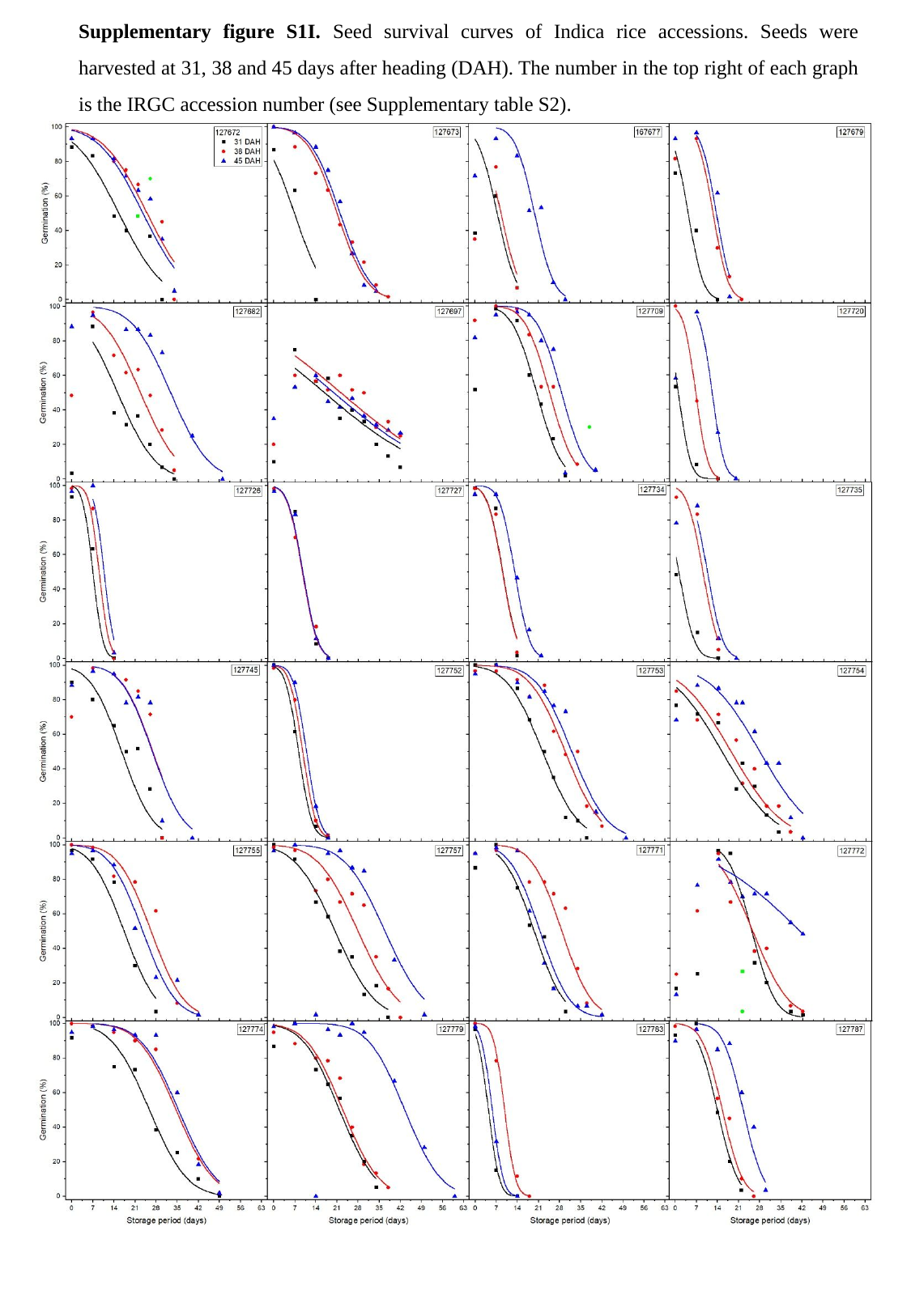

Supplementary figure S1I. Seed survival curves of Indica rice accessions. Seeds were harvested at 31, 38 and 45 days after heading (DAH). The number in the top right of each graph is the IRGC accession number (see Supplementary table S2).

## Slide 10
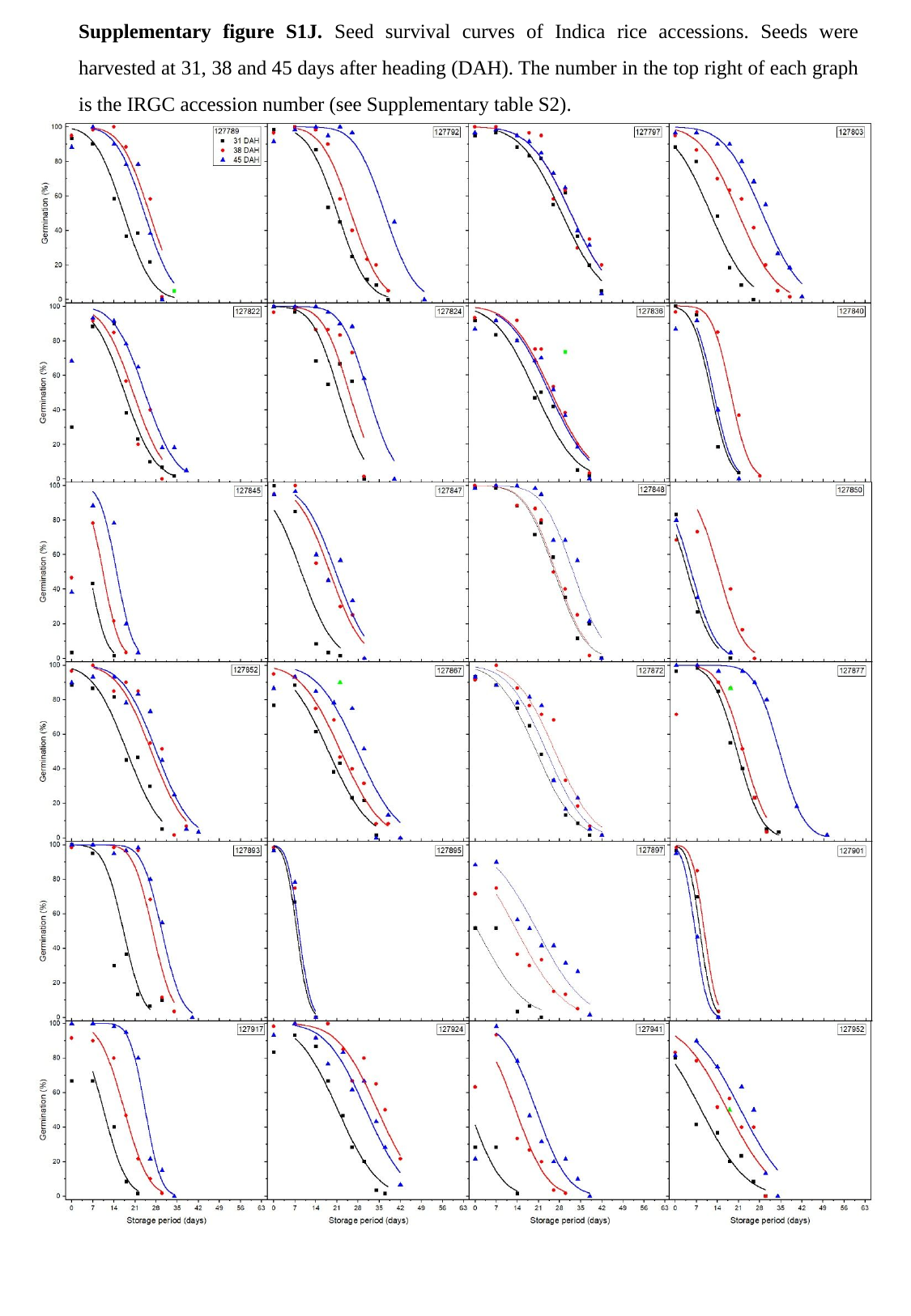

Supplementary figure S1J. Seed survival curves of Indica rice accessions. Seeds were harvested at 31, 38 and 45 days after heading (DAH). The number in the top right of each graph is the IRGC accession number (see Supplementary table S2).

## Slide 11
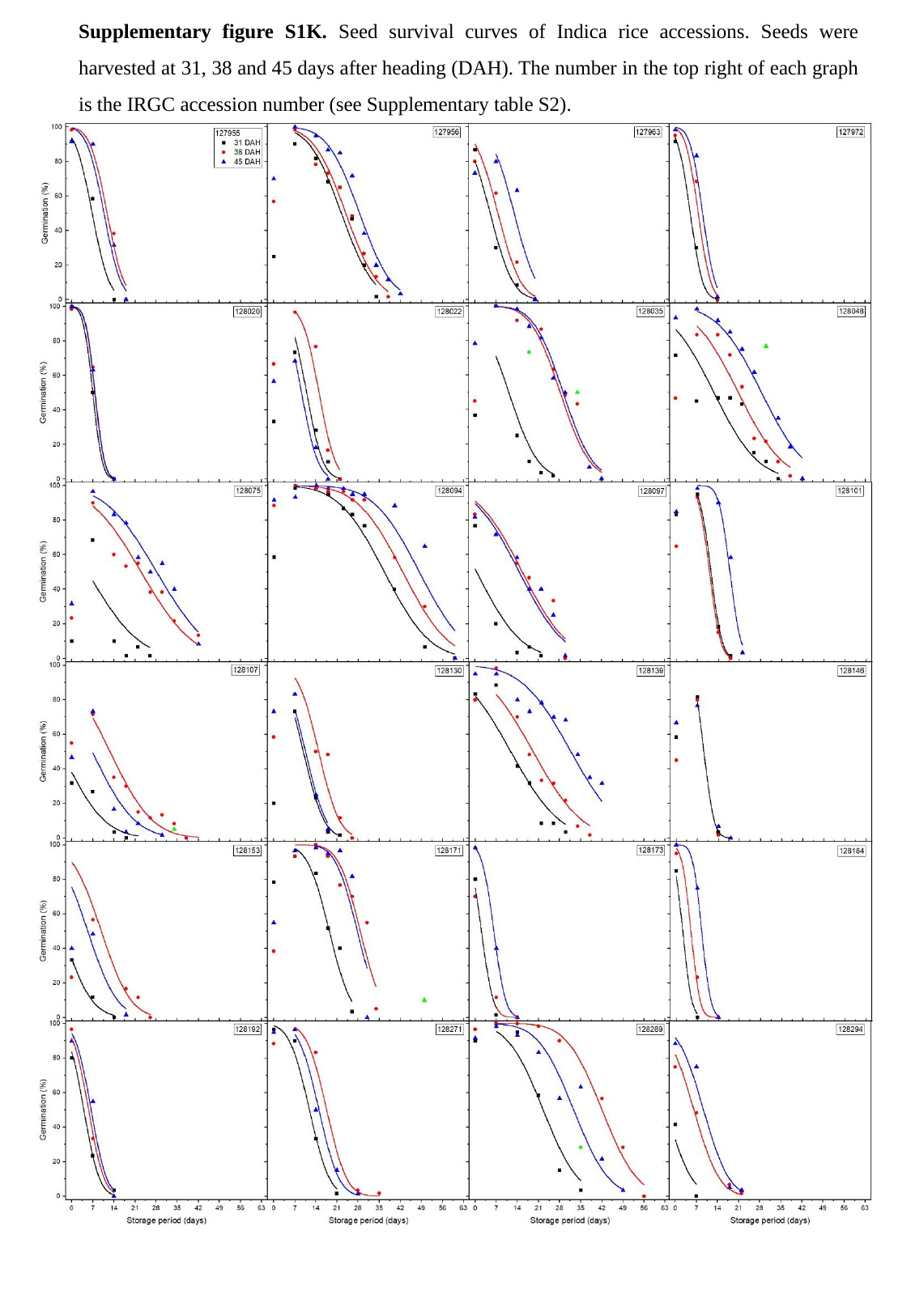

Supplementary figure S1K. Seed survival curves of Indica rice accessions. Seeds were harvested at 31, 38 and 45 days after heading (DAH). The number in the top right of each graph is the IRGC accession number (see Supplementary table S2).

## Slide 12
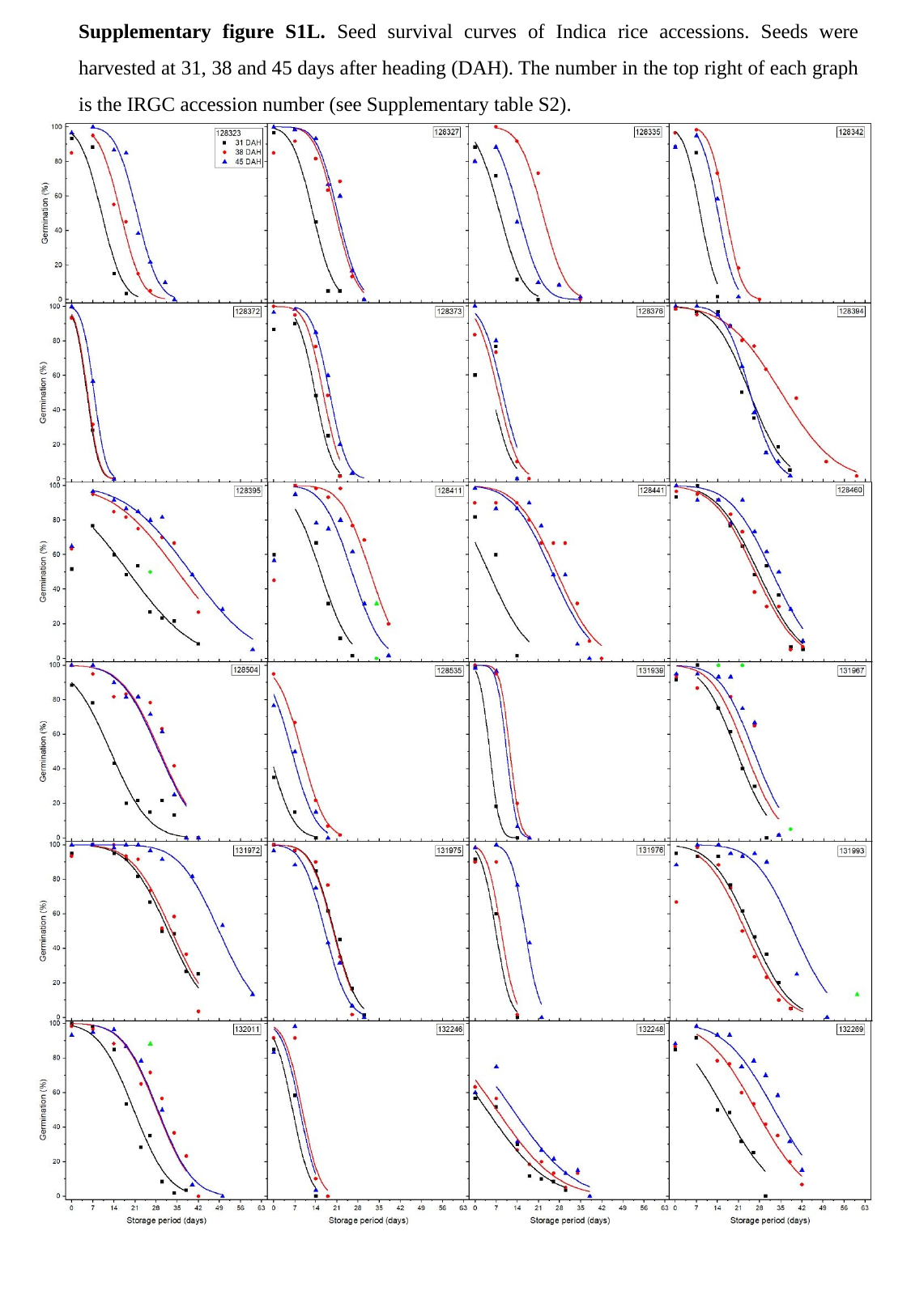

Supplementary figure S1L. Seed survival curves of Indica rice accessions. Seeds were harvested at 31, 38 and 45 days after heading (DAH). The number in the top right of each graph is the IRGC accession number (see Supplementary table S2).

## Slide 13
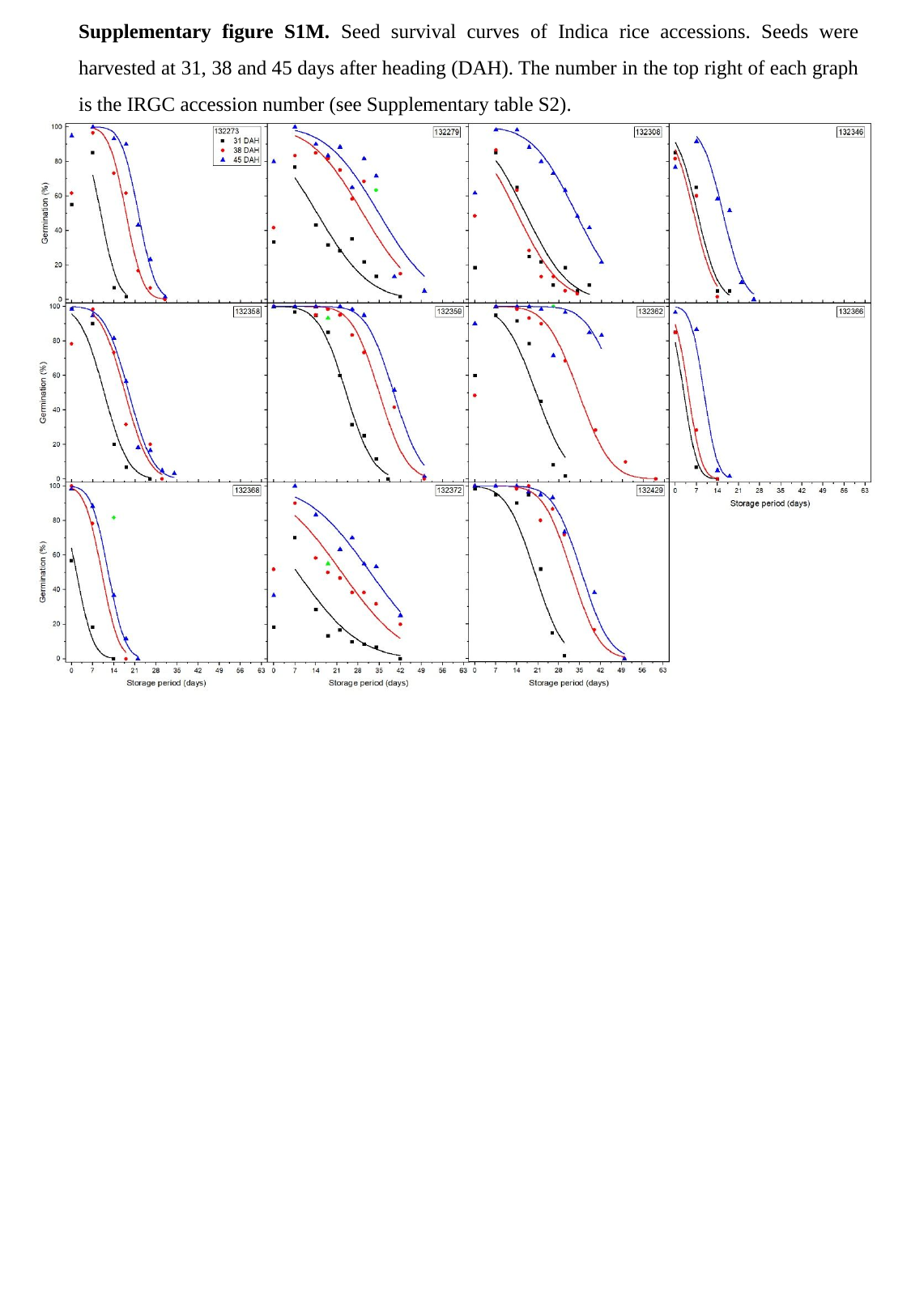

Supplementary figure S1M. Seed survival curves of Indica rice accessions. Seeds were harvested at 31, 38 and 45 days after heading (DAH). The number in the top right of each graph is the IRGC accession number (see Supplementary table S2).
