# Supplementary material for: Variation in seed longevity among diverse Indica rice varieties
Source: Ann Bot. 2019 Jun 10;124(3):447–60. doi: 10.1093/aob/mcz093 (PMC6798842; doi:10.1093/aob/mcz093)
Supplement: mcz093_suppl_Supplementary_Figure_S2 [file mcz093_suppl_supplementary_figure_s2.pptx]

## Slide 1
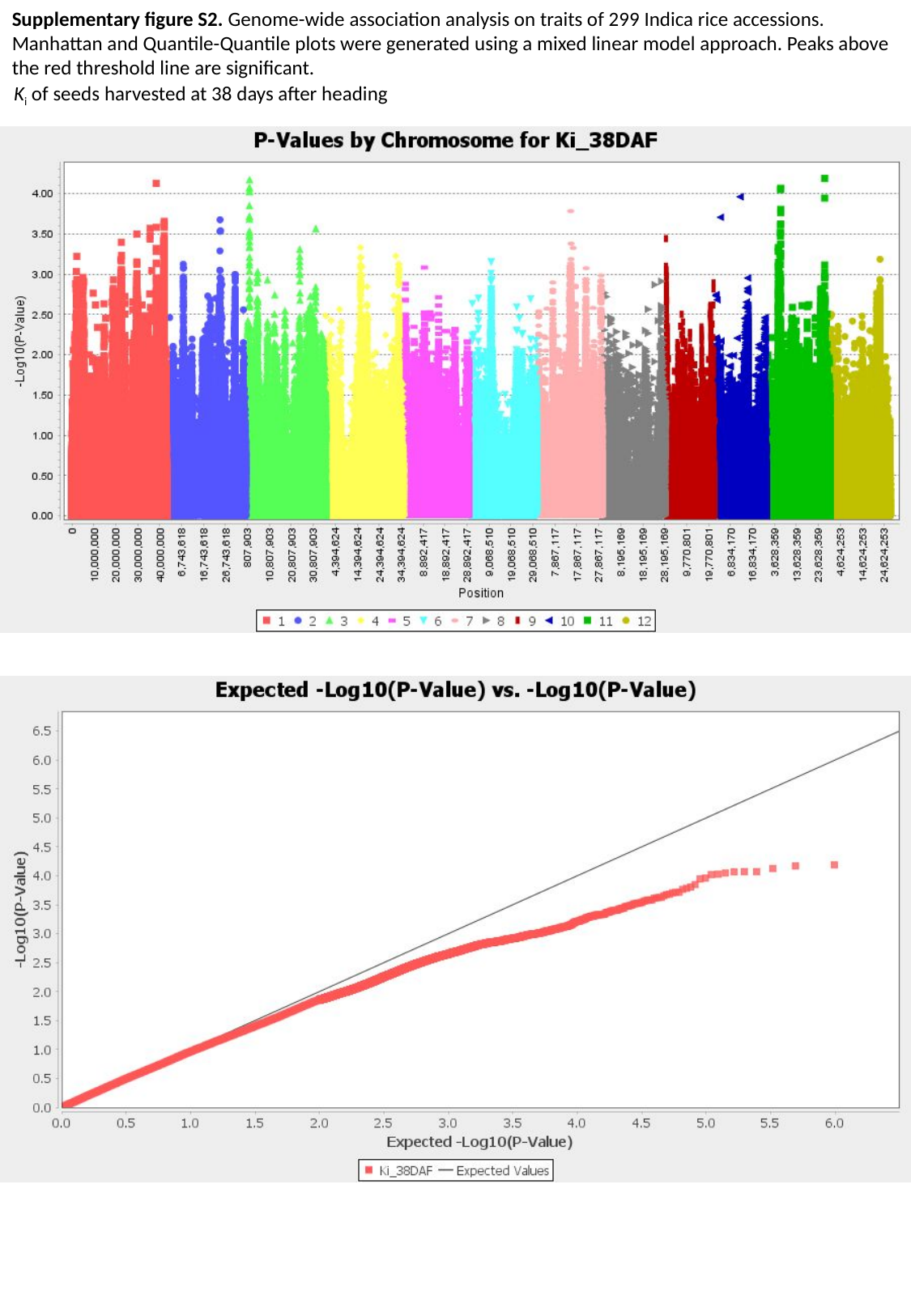

Supplementary figure S2. Genome-wide association analysis on traits of 299 Indica rice accessions. Manhattan and Quantile-Quantile plots were generated using a mixed linear model approach. Peaks above the red threshold line are significant.
Ki of seeds harvested at 38 days after heading

## Slide 2
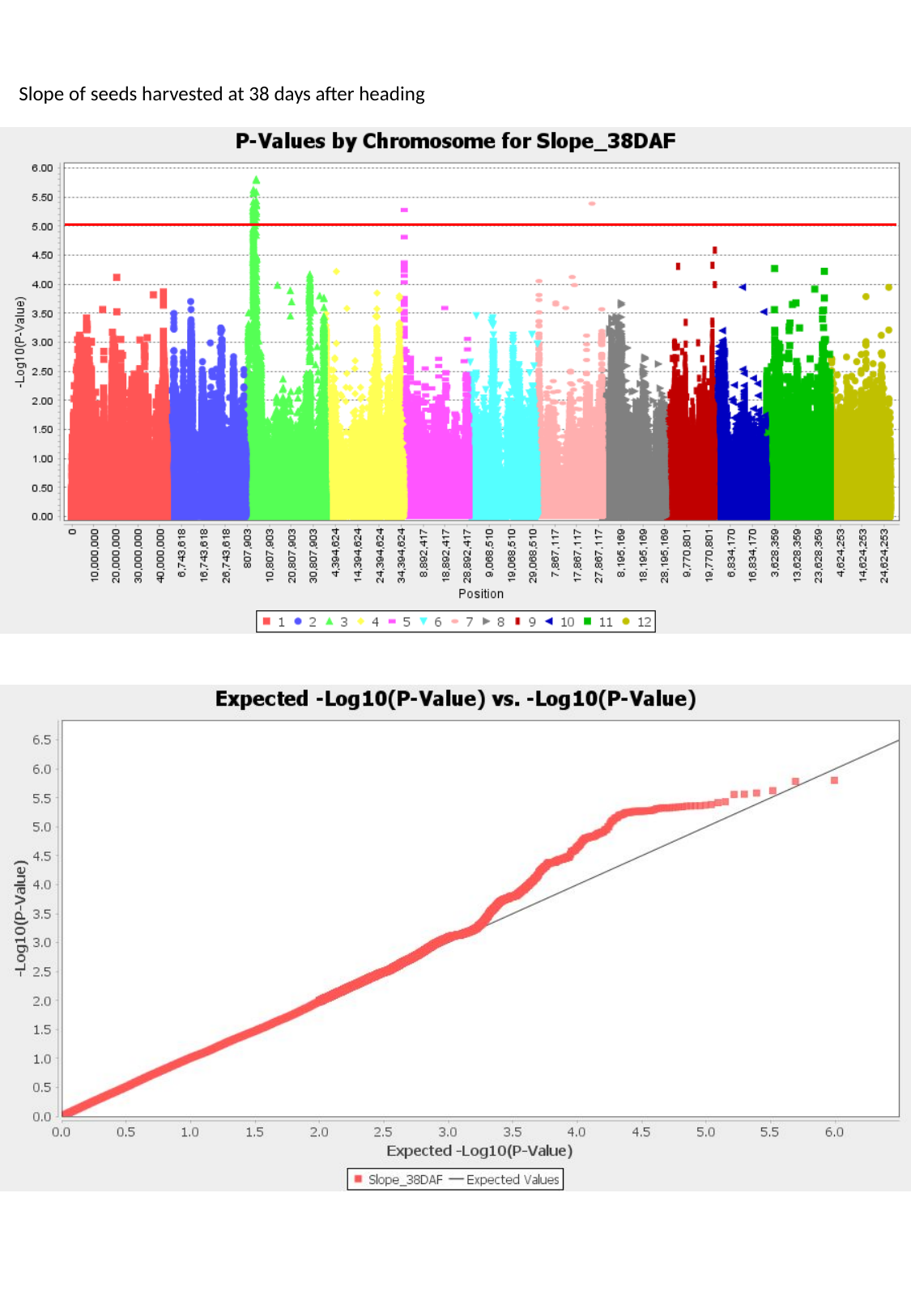

Slope of seeds harvested at 38 days after heading

## Slide 3
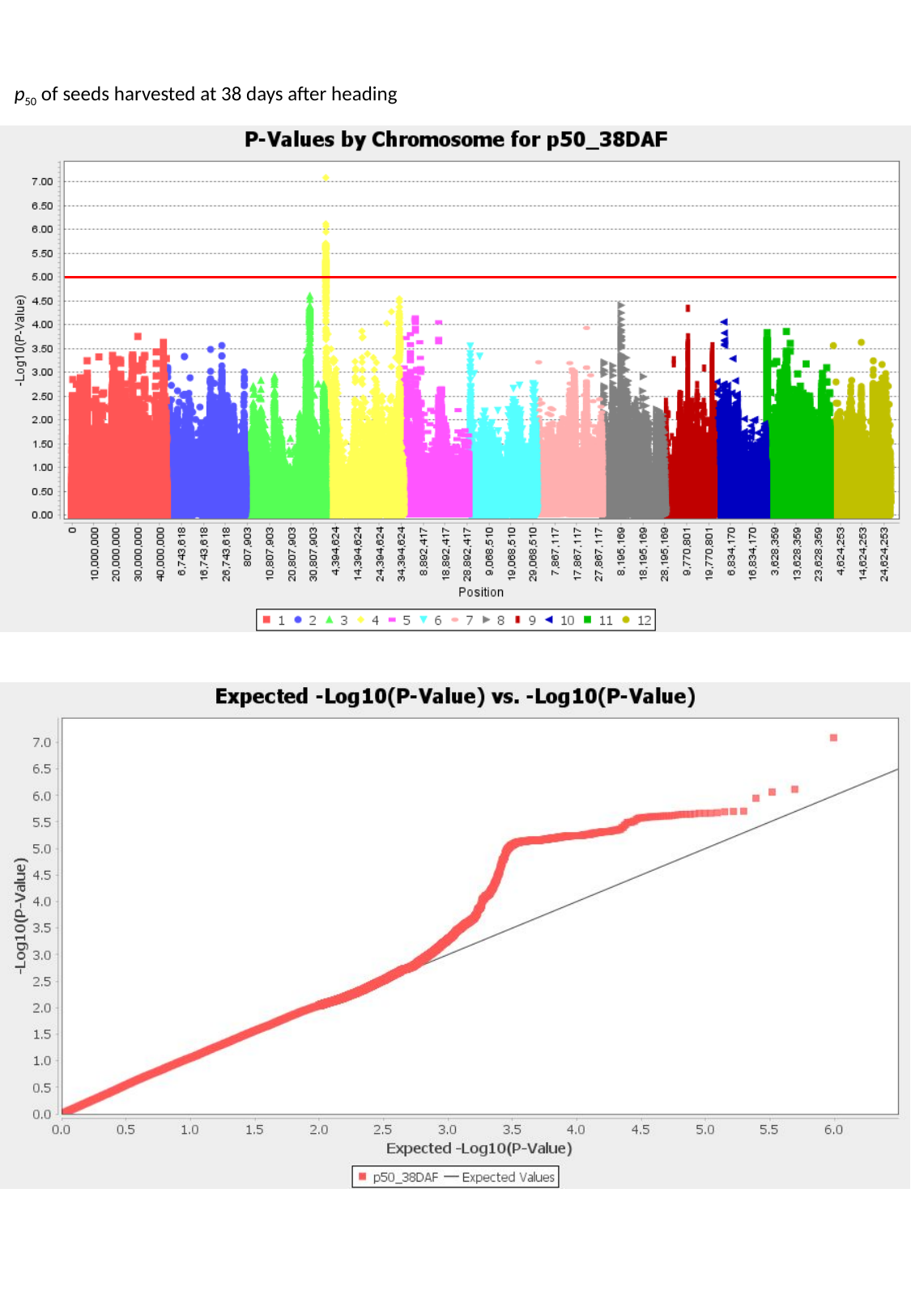

p50 of seeds harvested at 38 days after heading

## Slide 4
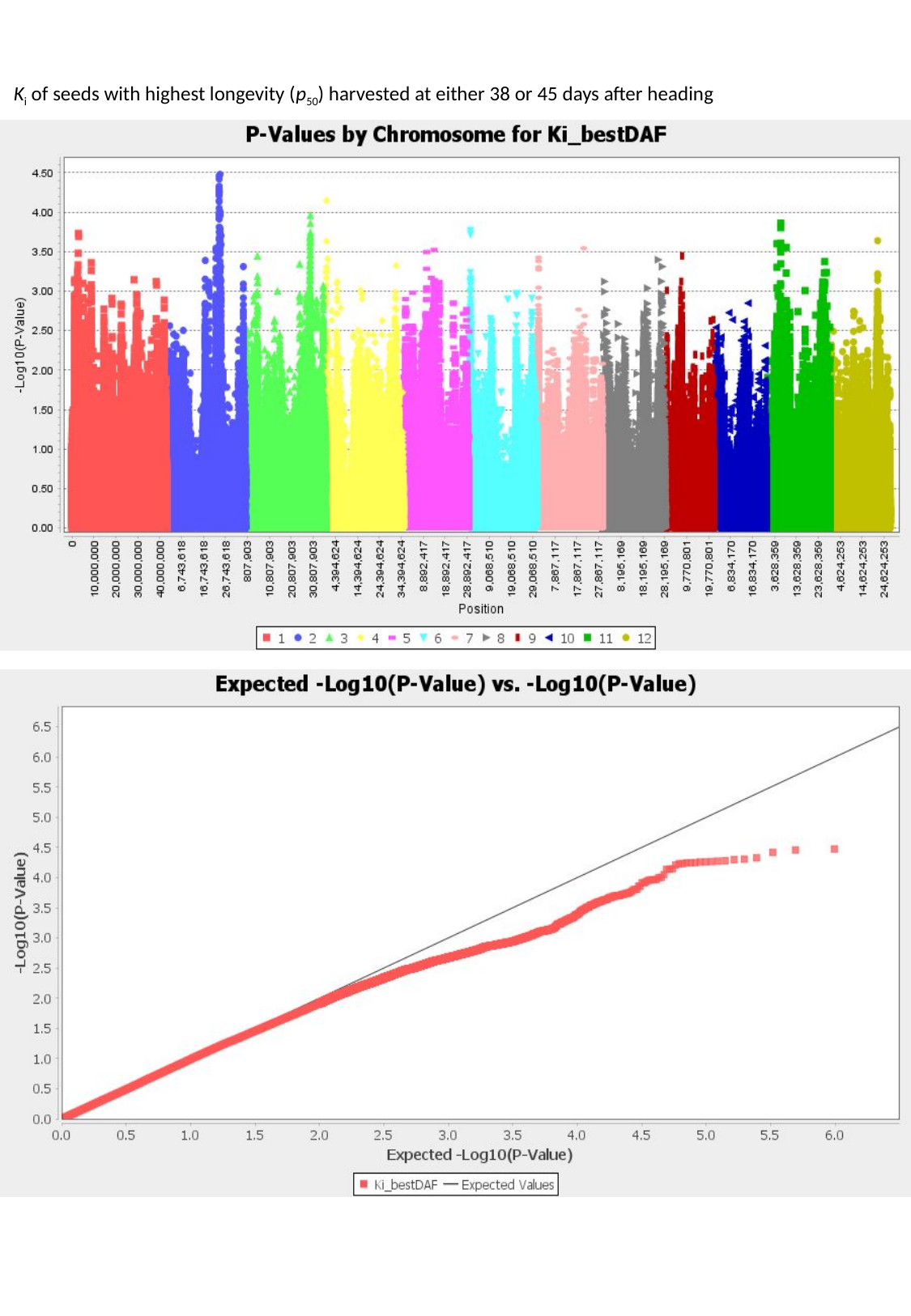

Ki of seeds with highest longevity (p50) harvested at either 38 or 45 days after heading

## Slide 5
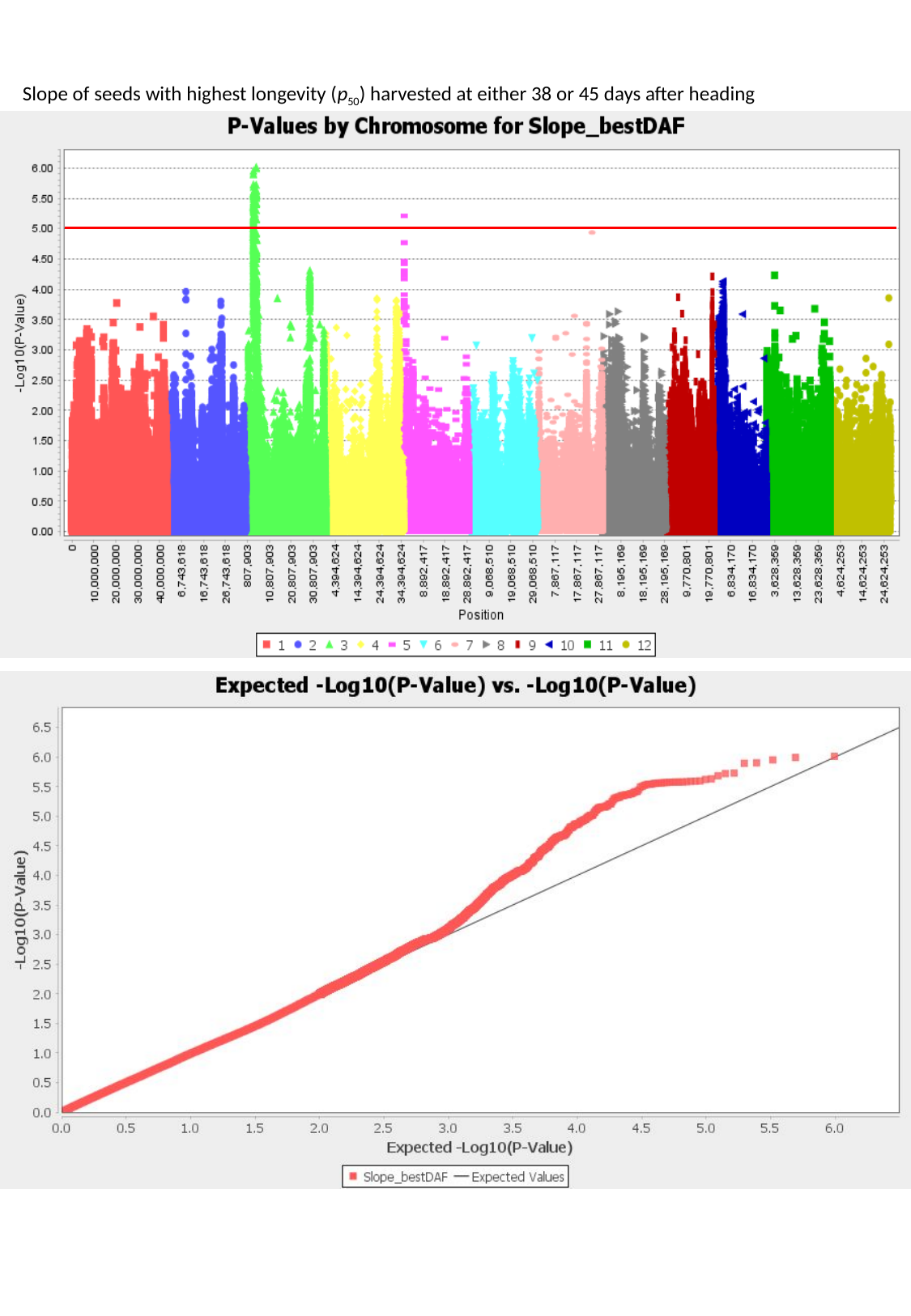

Slope of seeds with highest longevity (p50) harvested at either 38 or 45 days after heading

## Slide 6
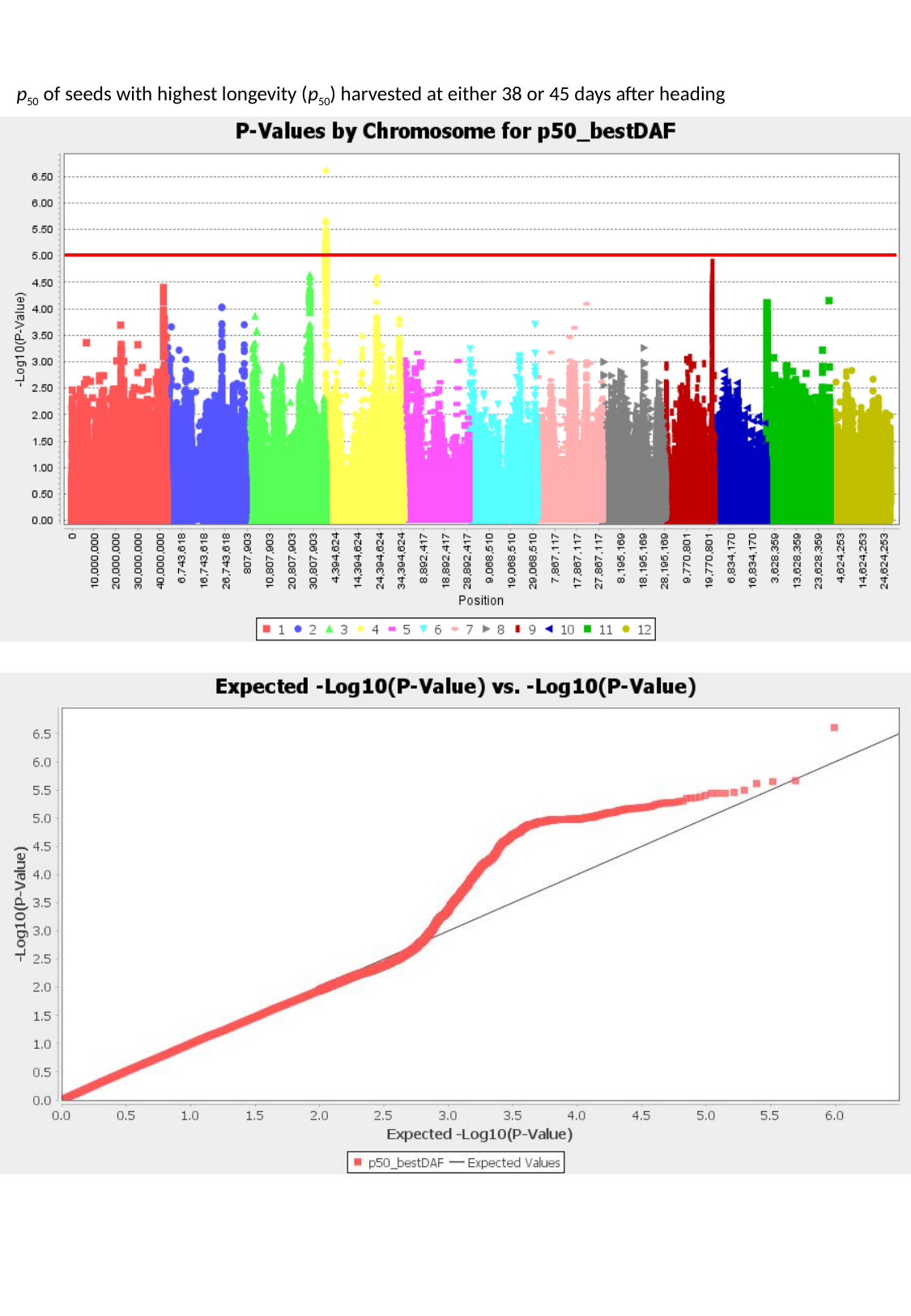

p50 of seeds with highest longevity (p50) harvested at either 38 or 45 days after heading

## Slide 7
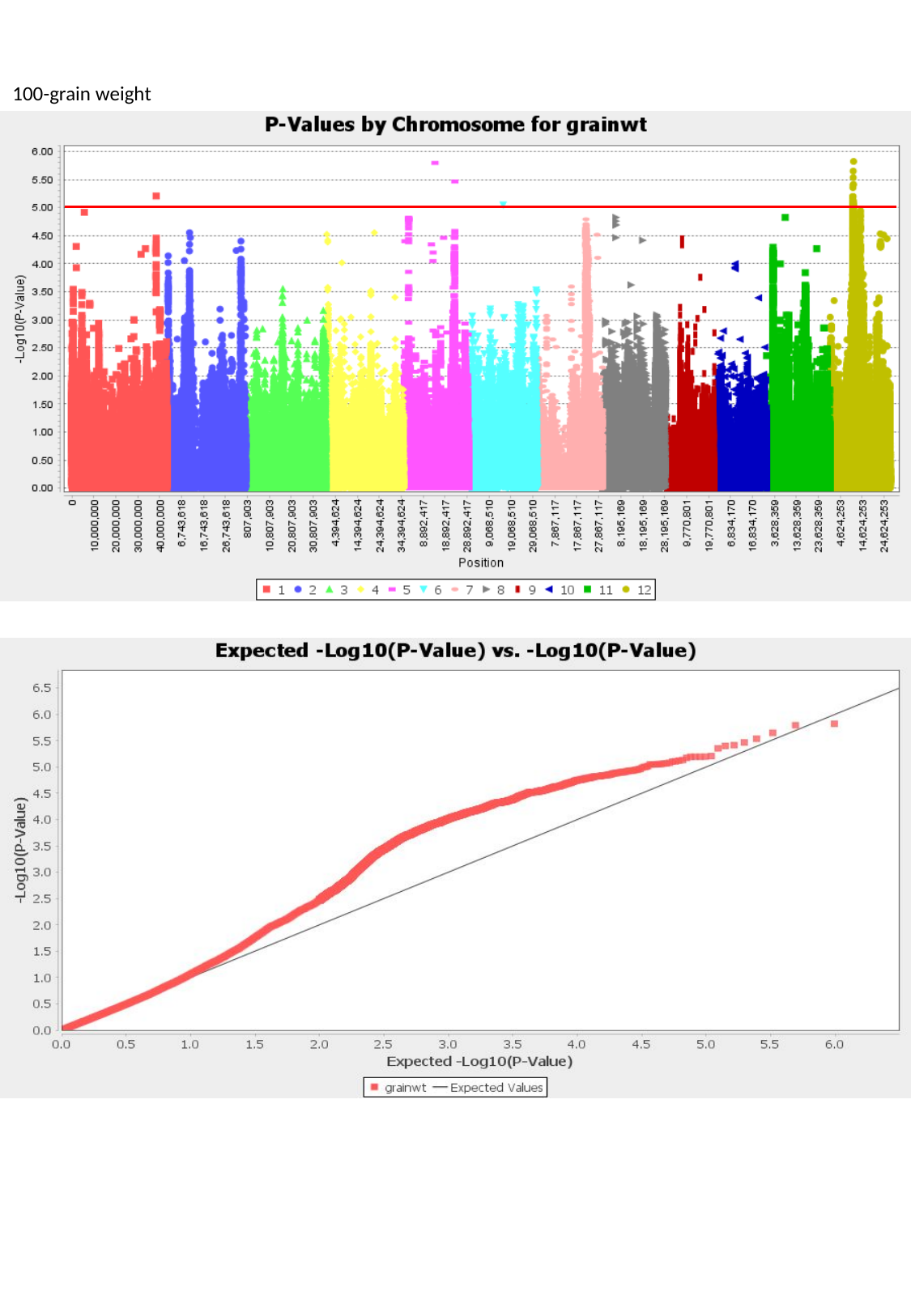

100-grain weight

## Slide 8
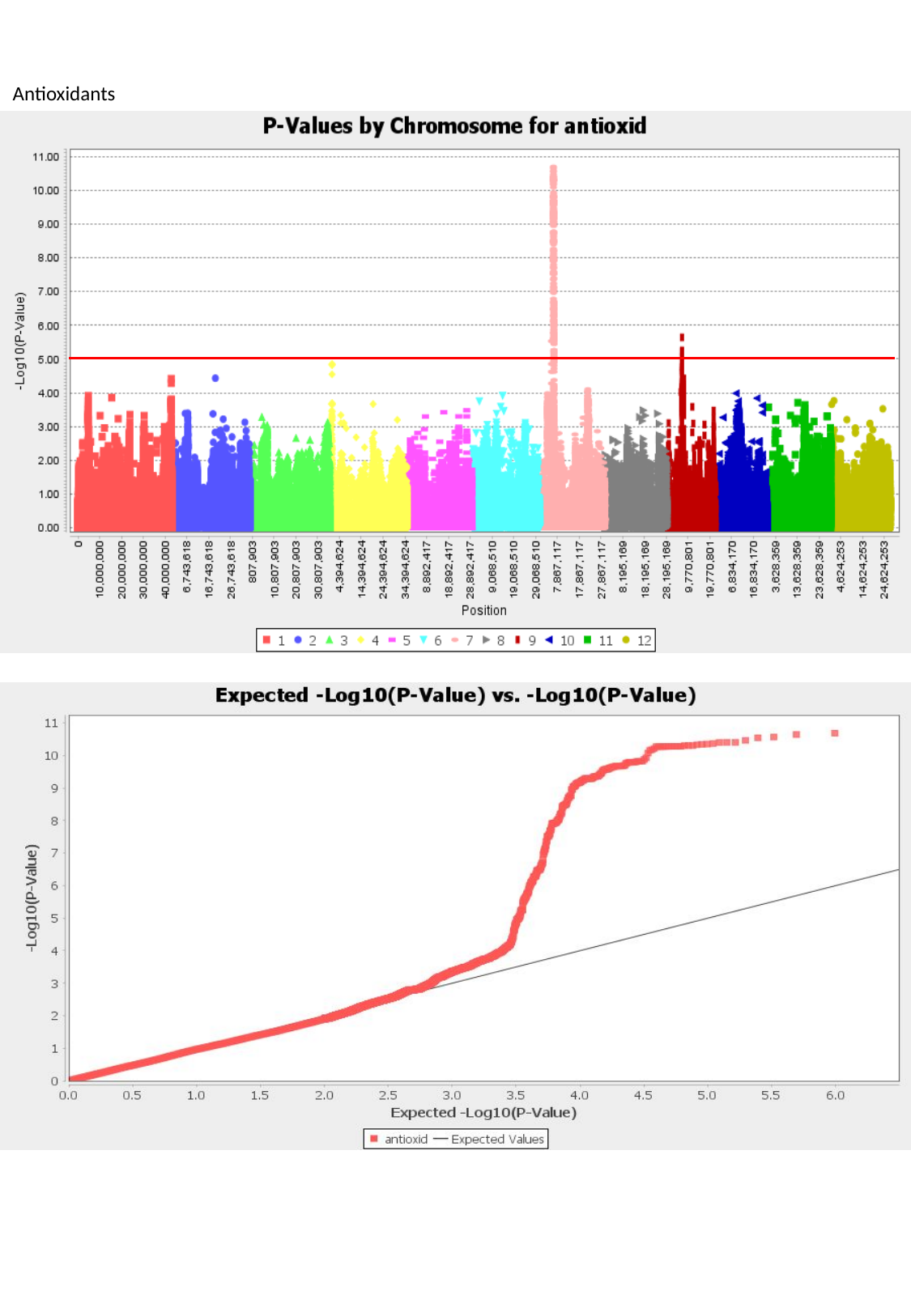

Antioxidants

## Slide 9
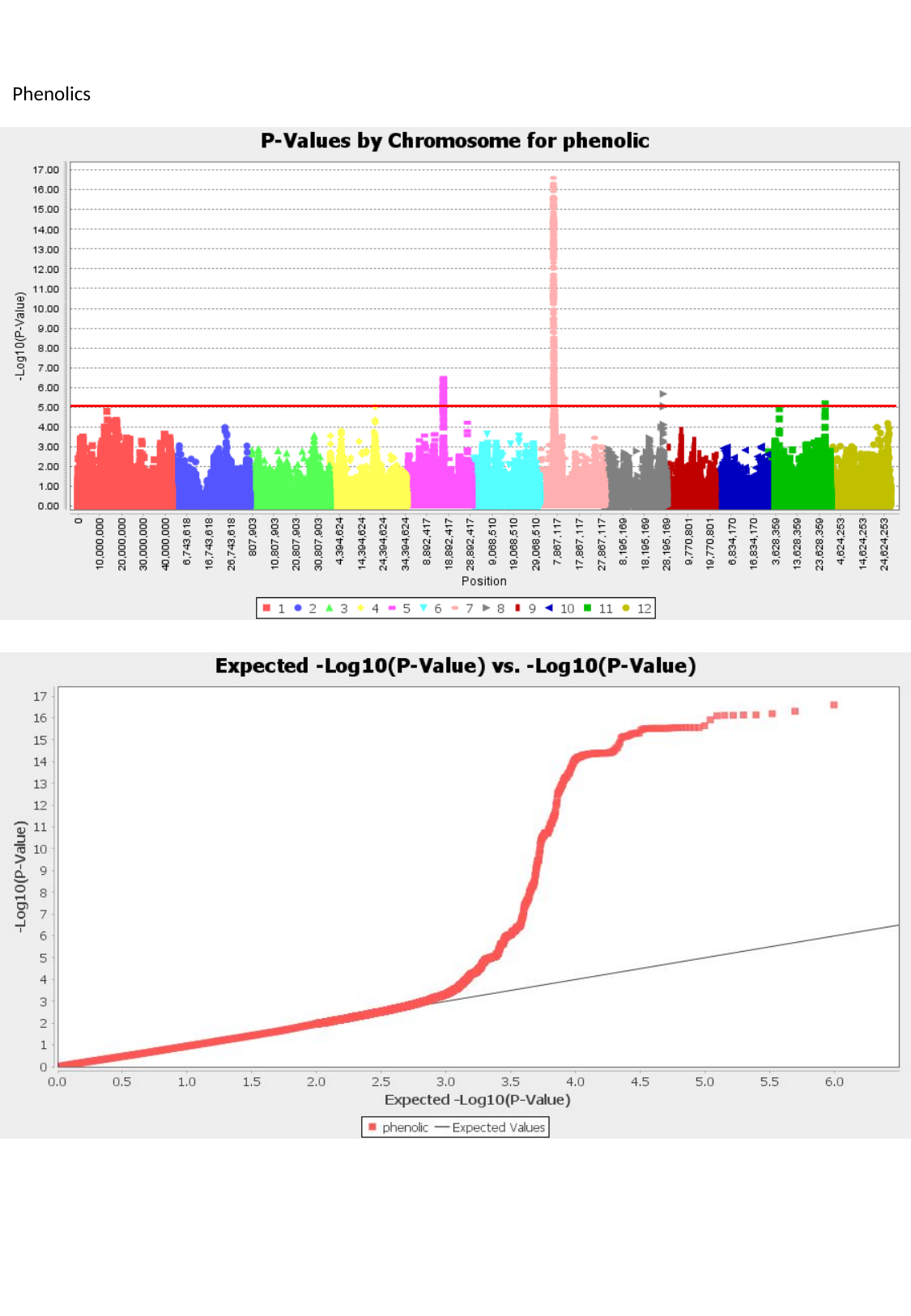

Phenolics
