# Supplementary material for: Variation in seed longevity among diverse Indica rice varieties
Source: Ann Bot. 2019 Jun 10;124(3):447–60. doi: 10.1093/aob/mcz093 (PMC6798842; doi:10.1093/aob/mcz093)
Supplement: mcz093_suppl_Supplementary_Table_S1 [file mcz093_suppl_supplementary_table_s1.docx]

**Table S1** Origin of the Indica rice accessions used in the seed longevity study.

| Continent | Region | Country | No. of accession |
| --- | --- | --- | --- |
| Africa | East Africa | Burundi | 1 |
|  |  | Kenya | 5 |
|  |  | Madagascar | 12 |
|  |  | Tanzania | 3 |
|  | Central Africa | Chad | 1 |
|  |  | Cameroon | 1 |
|  | North Africa | Egypt | 1 |
|  |  | Liberia | 1 |
|  | South Africa | Zambia | 1 |
|  | West Africa | Burkina Faso | 2 |
|  |  | Cote d'Ivoire | 2 |
|  |  | Gambia | 2 |
|  |  | Ghana | 1 |
|  |  | Guinea | 2 |
|  |  | Mali | 1 |
|  |  | Nigeria | 1 |
|  |  | Senegal | 9 |
|  |  | Sierra Leone | 1 |
| Asia | East Asia | China | 60 |
|  |  | Republic of Korea | 4 |
|  |  | Taiwan | 4 |
|  | Southeast Asia | Brunei | 1 |
|  |  | Cambodia | 6 |
|  |  | Indonesia | 8 |
|  |  | Laos | 20 |
|  |  | Malaysia | 1 |
|  |  | Myanmar | 15 |
|  |  | Philippines | 19 |
|  |  | Thailand | 22 |
|  |  | Vietnam | 1 |
|  | South Asia | Bangladesh | 17 |
|  |  | India | 48 |
|  |  | Nepal | 6 |
|  |  | Sri Lanka | 5 |
| Europe | Southeast Europe | Romania | 1 |
| North America | Caribbean | Dominican Republic | 1 |
|  | Central America | Guatemala | 1 |
|  |  | Mexico | 1 |
|  |  | Nicaragua | 1 |
| Oceania | Melanesia | Fiji | 1 |
| South America | South America | Brazil | 1 |
|  |  | Colombia | 4 |
|  |  | Ecuador | 2 |
|  |  | Paraguay | 1 |
| Unknown | | | 1 |
